# IRF2‐mediated upregulation of lncRNA HHAS1 facilitates the osteogenic differentiation of bone marrow‐derived mesenchymal stem cells by acting as a competing endogenous RNA

**DOI:** 10.1002/ctm2.429

**Published:** 2021-06-20

**Authors:** Guiwen Ye, Peng Wang, Zhongyu Xie, Jinteng Li, Guan Zheng, Wenjie Liu, Qian Cao, Ming Li, Shuizhong Cen, Zhaofeng Li, Wenhui Yu, Yanfeng Wu, Huiyong Shen

**Affiliations:** ^1^ Department of Orthopedics The Eighth Affiliated Hospital Sun Yat‐sen University Shenzhen P.R. China; ^2^ Center for Biotherapy The Eighth Affiliated Hospital Sun Yat‐sen University Shenzhen P.R. China; ^3^ Department of Orthopedics Sun Yat‐sen Memorial Hospital Sun Yat‐sen University Guangzhou P.R. China

**Keywords:** IRF2, long noncoding RNAs, mesenchymal stem cells, osteogenic differentiation

## Abstract

**Background:**

Mesenchymal stem cells (MSCs) are the major source of osteoblasts. Long noncoding RNAs (lncRNAs) are abundantly expressed RNAs that lack protein‐coding potential and play an extensive regulatory role in cellular biological activities. However, the regulatory network of lncRNAs in MSC osteogenesis needs further investigation.

**Methods:**

QRT‐PCR, western blot, immunofluorescence, and immunohistochemistry assays were used to determine the levels of relevant genes. The osteogenic differentiation capability was evaluated by using Alizarin Red S (ARS) staining, alkaline phosphatase activity assays, hematoxylin & eosin staining or micro‐CT. RNA fluorescence in situ hybridization (FISH) and RNAscope were used to detect HHAS1 expression in cells and bone tissue. A microarray assay was performed to identify differentially expressed microRNAs. RNA immunoprecipitation and RNA pull‐down were used to explore the interactions between related proteins and nucleic acids.

**Results:**

The level of lncRNA HHAS1 increased during bone marrow‐derived MSC (BMSC) osteogenesis and was positively related to the levels of osteogenic genes and ARS intensity. HHAS1 was located in both the cytoplasm and the nucleus and was expressed in human bone tissue. HHAS1 facilitated BMSC osteogenic differentiation by downregulating miR‐204‐5p expression and enhancing the level of RUNX family transcription factor 2 (RUNX2). In addition, interferon regulatory factor 2 (IRF2) was increased during BMSC osteogenic differentiation and interacted with the promoter of HHAS1, which resulted in the transcriptional activation of HHAS1. Furthermore, IRF2 and HHAS1 helped improve bone defect repair in vivo.

**Conclusions:**

Our study identified a novel lncRNA, HHAS1, that facilitates BMSC osteogenic differentiation and proposed a role for the IRF2/HHAS1/miR‐204‐5p/RUNX2 axis in BMSC osteogenesis regulation. These findings help elucidate the regulatory network of BMSC osteogenesis and provide potential targets for clinical application.

## Graphical abstract

Graphical Highlights

1. Under the condition of osteogenic induction, the level of IRF2 is increased and promotes the transcription of lncRNA HHAS1.

2. HHAS1 sponges miR‐204‐5p by acting as a ceRNA, thereby protecting RUNX2 mRNA from translation inhibition and facilitating BMSC osteogenesis.

3. Modulating IRF2 or HHAS1 can regulate BMSC osteogenic differentiation and help improve bone defect impair.

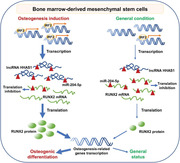

**Abbreviations**
AGO2: argonaute RISC catalytic component 2
ALP: alkaline phosphatase
ARS: Alizarin Red S
ATF2: activating transcription factor 2
BMSCs: bone marrow‐derived mesenchymal stem cells
ceRNA: competing endogenous RNA
ColI: collagen I
CPAT: Coding Potential Assessment Tool
CPC: Coding Potential Calculator
DAPI: 40,6‐diamidino‐2‐phenylindole
DMEM: Dulbecco's modified Eagle's medium
FBS: fetal bovine serum
FISH: RNA fluorescence in situ hybridization
FOXP3: forkhead box P3
GAPDH: glyceraldehyde‐3‐phosphate dehydrogenase
H19: H19 imprinted maternally expressed transcript
HHAS1: lncRNA HHIP antisense RNA 1
hnRNPK: heterogeneous nuclear ribonucleoprotein K
HOTAIR: HOX transcript antisense RNA
HOXB7: homeobox B7
IRF2: interferon regulatory factor 2
lncRNA: long noncoding RNA
MALAT1: metastasis‐associated lung adenocarcinoma transcript 1
MEG3: maternally expressed 3
miRNA: microRNA
MOI: multiplicity of infection
MSCs: mesenchymal stem cells
MUT: mutant
NC: negative controls
OCN: osteocalcin
OM: osteogenic differentiation medium
PBS: phosphate‐buffered saline
RIP: RNA immunoprecipitation
RISC: RNA‐induced silencing complex
RUNX2: RUNX family transcription factor 2
siRNA: small interfering RNA
TF: transcription factor
WT: wild‐type
XBP1: X‐box binding protein 1
YAP: Yes1‐associated transcriptional regulator
YY1: YY1 transcription factor

## 1 BACKGROUND

Mesenchymal stem cells (MSCs) are a type of stromal cell with self‐renewal ability and multilineage differentiation potential.^1^ MSCs can be derived from various kinds of tissues, such as menstrual blood, bone marrow and fat and can differentiate into osteoblasts, adipocytes and chondrocytes.^1^ In recent decades, MSCs have been considered the major source of osteoblasts and are thought to participate in bone formation and repair.^2^ MSCs with aberrant osteogenic differentiation ability have been found in various diseases and may participate in pathogenesis.^3^ In addition, numerous studies have attempted to determine the mechanism of osteogenic differentiation in MSCs and have explored their potential clinical application value.^3^ However, further investigation of the regulatory network associated with osteogenic differentiation in MSCs is required.

Long noncoding RNAs (lncRNAs) are a special type of transcript containing more than 200 nucleotides and lacking the ability to encode protein.^4^ They play an extensive regulatory role in cellular biological activities and have attracted substantial attention in the past few years.^5^ LncRNAs can regulate cellular functions at the transcriptional, translational and post‐translational levels.^4^ Bone metabolism is mainly regulated by osteoclasts, which play a role in bone resorption, and osteoblasts, which mediate new bone formation.^6^ Recently, studies have reported that lncRNAs are involved in diseases related to bone metabolism and are critical for the differentiation of MSCs.^7^ In addition, we have previously shown that substantial changes in lncRNA profiles occur in MSCs during osteogenic differentiation.^8^ However, the underlying mechanism by which lncRNAs regulate MSC osteogenic differentiation is still not clear. Characterizing the regulatory network of lncRNAs in MSC osteogenic differentiation may help provide new insight into bone metabolism and potential intervention targets for bone‐related diseases.

MicroRNAs (miRNAs) are small RNAs that play important roles in gene regulation by binding to messenger RNAs (mRNAs) and suppressing protein translation.^9^ Numerous studies have revealed that miRNAs play a key role in regulating stem cell differentiation.^10^, ^11^ MiR‐204‐5p is a miRNA with multiple functions in proliferation, apoptosis, and differentiation, among others.^12^, ^13^, ^14^ Usually, miRNAs can be sponged by lncRNAs acting as competing endogenous RNAs (ceRNAs), thus protecting downstream mRNAs from degradation or translational inhibition.^15^ The role of the lncRNA‐miRNA‐mRNA regulatory network in MSC osteogenic differentiation deserves further study.

Transcription factors (TFs) are vital components of the transcriptional regulation machinery and play a crucial role in cell differentiation.^16^ Commonly, TFs interact with gene promoters and promote or block gene transcription.^16^ The TF interferon regulatory factor 2 (IRF2) has been reported to participate in cancer growth and immune cell function.^17^ Whether IRF2 is involved in the regulatory network of MSC differentiation has rarely been reported.

In this study, we explored the lncRNAs participating in the osteogenesis of bone marrow‐derived MSCs (BMSCs) and identified an lncRNA, HHAS1, that was increased during BMSC osteogenic differentiation and promoted BMSC osteogenesis in vitro and in vivo. Mechanistically, HHAS1 sponged miR‐204‐5p by acting as a ceRNA, thereby upregulating the expression of RUNX family TF 2 (RUNX2). Additionally, the increase in IRF2 expression resulted in the transcriptional activation of HHAS1 during osteogenesis. Overall, we identified a new lncRNA responsible for BMSC osteogenic differentiation and characterized the IRF2/HHAS1/miR‐204‐5p/RUNX2 pathway, which helps to elucidate the regulatory network of BMSC osteogenesis and may provide new insights for clinical application.

## 2 METHODS

### 2.1 Ethical statement

The approval of this study was got from The Eighth Affiliated Hospital, Sun Yat‐sen University, China. All subjects have been informed of the study procedure and potential risks and the informed consent have been signed.

### 2.2 Isolation and expansion of BMSCs

The isolation and expansion of BMSCs were performed as described.^18^ Briefly, bone marrow was extracted from ilium derived from humans and mixed with the separation solution. The mixture was incubated for approximately 40 min and centrifuged at 2,500 × g for 30 min. Subsequently, BMSCs were collected and cultured in medium composed of 90% Dulbecco's modified Eagle's medium (DMEM, Gibco) and 10% fetal bovine serum (FBS, Zhejiang Tianhang Biotechnology). Three days later, the detached cells were removed by medium replacement. After that, the culture medium was replaced every 3 days. When the BMSCs reached 80%–90% confluence, they were digested by trypsin and divided into two new culture flasks. Fifteen BMSC lines were used in this study, and the BMSCs used in subsequent experiments were at passage 4. BMSCs at 0.5 × 10^5^ cells/well were seeded in 12‐well plates for osteogenic induction and further evaluation.

### 2.3 Osteogenic differentiation induction

The osteogenic differentiation medium (OM) consisted of 10% FBS DMEM supplemented with penicillin‐streptomycin (100 IU/ml), dexamethasone (0.1 μM), β‐glycerol phosphate (10 mM) and ascorbic acid (50 μM). Overall, 2 ml/well of OM was used and the OM was replaced every 3 days until the BMSCs were used in subsequent assays.

### 2.4 ARS staining assay

BMSCs were fixed, and 500 μl 1% Alizarin Red S (ARS) was added to the plate wells. After 15 min, the staining solution was discarded, and the plate wells were washed with phosphate‐buffered saline (PBS) three times. The results were obtained with an optical microscope. After that, cetylpyridinium chloride monohydrate solution (Sigma‐Aldrich) was added to extract the dye, and the absorbance (562 nm) was measured with a multiscan spectrophotometer.

### 2.5 ALP assay

For alkaline phosphatase (ALP) staining, 4% paraformaldehyde was added to the plate wells, and the BMSCs were fixed for 15 min. A BCIP/NBT ALP kit (Beyotime, China) was used in accordance with the manufacturer's directions. The results were obtained with an optical microscope. For the ALP activity measurement, BMSCs were washed with PBS buffer three times and lysed by RIPA buffer. Then, the protein concentration was detected by a BCA Protein Assay Kit (CWBIO, CW0014S), and ALP activity was measured using ALP activity detection kits (Jiancheng Biotech, China) and calculated as manufacturer's directions.

### 2.6 RNA extraction and quantitative real‐time PCR

A TRIzol solution (TaKaRa) was applied to extract total RNA of BMSCs. A PrimeScript RT Kit (TaKaRa) was used to reverse transcribe RNA into cDNA. Then, quantitative real‐time PCR (qPCR) was performed by using TB Green Premix Ex Taq II (TaKaRa) as the manufacturer's directions. Gene expression was examined using a real‐time PCR System. The relative gene expressions were calculated with GAPDH as the reference gene. The relevant primers are shown in Table S1.

### 2.7 Rapid amplification of cDNA ends (RACE)

A SMARTer RACE amplification kit (Clontech, USA) was used as described previously.^7^ First, total RNA of BMSCs was isolated and first‐strand cDNA for 3′‐ and 5′‐RACE experiments was synthesized. Then, amplification was performed on a PCR amplifier according to the instructions. The specific bands were collected, and the products sequences were determined. The relevant primers are shown in Table S2.

### 2.8 RNA fluorescence in situ hybridization

The probes were provided by RiboBio (Guangzhou, China), and the FISH assay was performed by using a FISH kit (RiboBio, C10910) in accordance with the directions. First, BMSCs were fixed followed by prehybridization in PBS. Then, the cells were hybridized at 37°C for 30 min. Finally, the cells were stained with 4′,6‐diamidino‐2‐phenylindole (DAPI), and the probe signals were captured under a fluorescence microscope.

### 2.9 Cell cytoplasmic/nuclear fractionation

Fractionation of the cell cytoplasm/nucleus was performed using a PARIS kit (Thermo Fisher). Cytoplasmic and nuclear RNA from BMSCs was separated and reverse transcribed. The HHAS1 levels were evaluated by qPCR, and the percentages of nuclear and cytoplasmic RNA were analyzed.

### 2.10 RNA interference

Small interfering RNAs (siRNAs) targeting HHAS1, RUNX2, IRF2, and YY1 and the inhibitors and mimics of miR‐204‐5p and miR‐3529‐3p were purchased from GenePharma (Suzhou, China). When the seeded BMSCs reached 70%–90% confluence, transfections were performed by using Opti‐MEM and Lipofectamine RNAiMAX (Thermo Fisher) according to the directions. Then, the BMSCs were collected to measure the interference efficiency after 48 h or directly used in other experiments. The relevant sequences are listed in Table S3.

### 2.11 Lentivirus construction and infection

Full‐length HHAS1, RUNX2, and IRF2 overexpression lentiviruses were constructed on GFP‐loaded lentiviral vectors and purchased from GenePharma (Suzhou, China). For infection, the amount of lentivirus at a multiplicity of infection of 50 and 5 mg/ml polybrene was added to the culture medium. The medium was replaced after 24 h. Then, the transfection efficiency was detected by fluorescence microscopy and qPCR after 72 h or directly used in experiments.

### 2.12 Western blot

The experiment was performed as previously described.^14^ First, RIPA buffer was prepared, and cells were lysed for 30 min on ice. Then, the cell lysates were collected and centrifuged at 14,000 rpm for 30 min at 4°C. After that, the protein concentrations were measured, and equal amounts of proteins were mixed with loading buffer. The proteins were separated via electrophoresis and transferred to polyvinylidene fluoride membranes (Millipore, IPVH0010). Then the membranes were blocked with 5% non‐fat milk solution for 1 h. Thereafter, the membranes were incubated with primary antibodies followed by secondary antibodies. Finally, protein levels were detected using Chemiluminescent HRP Substrate (Millipore, WBKLS0500) and analyzed with ImageJ.

The following antibodies were used for western blotting: anti‐Osterix (Abcam, ab209484, 1:800); anti‐OCN (Abcam, ab93876, 1:800); anti‐AGO2 (Abcam, ab186733, 1:1500); anti‐RUNX2 (Cell Signaling Technology, 12556S, 1:1000); anti‐Collagen I (ColI; Abcam, ab260043, 1:1500); anti‐IRF2 (Abcam, ab124744, 1:2000); anti‐YY1 (Abcam, ab109228, 1:2000); anti‐GAPDH (CWBIO, CW0100M, 1:3000); and HRP‐conjugated secondary antibodies (Boster, 1:5000).

### 2.13 Immunofluorescence

First, the BMSCs were fixed and permeabilized. Subsequently, the cells were blocked with goat serum for 30 min and incubated with anti‐ColI (Abcam, ab260043, 1:250) at 4°C for 16 h. After that, the cells were incubated with fluorophore‐labeled secondary antibodies (Cell Signaling Technology, 4412S, 1:500) for 1 h and counterstained with DAPI for 15 min. The fluorescence signals were obtained with a fluorescence microscope.

### 2.14 Bone formation assay in vivo

The experiment was performed as described.^8^ First, BMSCs were infected with the overexpression lentivirus or control lentivirus and cultured in OM for 5 days. Then, the cells were transferred to scaffolds consisting of hydroxyapatite/tricalcium phosphate (HA/TCP; Zimmer). Subsequently, the loaded scaffolds were transplanted under the dorsal skin of nude mice (Laboratory Animal Center of Sun Yat‐Sen University, China). Eight weeks later, the implants were extracted followed by section preparation.

### 2.15 Staining of tissue sections

The sections from the bone‐formation assay were deparaffinized by xylene and hydrated by ethanol before staining. For hematoxylin & eosin (H&E) staining, the prepared slices were stained with hematoxylin for 5 min and washed in 70% alcohol containing 1% HCl, followed by eosin staining for 3 min. For Masson's trichrome staining, a Masson's trichrome staining kit (Sigma‐Aldrich) was used in accordance with the manufacturer's directions. For immunohistochemical staining, an anti‐OCN antibody (Abcam, ab93876) was used to assess osteogenesis. After the staining procedures, the sections were sealed and observed under an optical microscope.

### 2.16 RNA immunoprecipitation

An EZ‐Magna RNA immunoprecipitation (RIP) Kit (Millipore, 17–701) was used according to the manufacturer's instructions. Briefly, 5 × 10^6^ BMSCs were lysed and magnetic beads conjugated with anti‐AGO2 (Abcam, ab186733, 1:50) or negative control IgG (Cell Signaling Technology, 2729S, 1:50) were added. The immunoprecipitated RNAs and the input RNAs were extracted and subjected to qPCR followed by electrophoresis to detect the expression of HHAS1.

### 2.17 RNA pull‐down

For the pull‐down assay, a TranscriptAid T7 Kit (Thermo Fisher, K0441) and a RNA‐Protein Pull‐Down Kit (Thermo Fisher, 20164) were used in accordance with the instructions. Briefly, HHAS1 and antisense transcripts were transcribed and biotin‐labeled. Then, the labeled RNAs were incubated with BMSC lysates, and the pulled‐down proteins were extracted for further detection.

### 2.18 Luciferase reporter assay

The relevant sequences were synthesized and constructed to the pMIR‐Report luciferase plasmids vector. The promoter sequences were synthesized and inserted into the pGL4 vector by Obio Technology (Shanghai, China). 293T cells (2 × 10^5^ cells/well) were seeded in six‐well plates, and transfection was performed using Lipofectamine 3000 (Invitrogen, L3000001) followed by transfection of siRNAs or miRNA mimics or treatments. Then, the luciferase activities were measured using a Dual‐Luciferase Reporter kit (Promega, E1910) in accordance with the directions. The relative luciferase intensity is expressed as the ratio of firefly luciferase activity to Renilla luciferase activity.

### 2.19 RNAscope in situ hybridization assay

Human femoral heads were collected from patients with femoral neck fractures. An RNAscope Detection Kit from Advanced Cell Diagnostics (ACD) was used for RNAscope detection. The specimens were fixed with 10% neutral formalin and decalcified by Preserve Rapid Decalcification Solution (Pursuit Bio, Beijing, China, 0500‐0050‐500). Then, the bone tissues were embedded, and the slices were prepared. The HHAS1 probe for RNAscope detection was provided by ACD. The detection of HHAS1 in bone tissue was performed in accordance with the instructions under a light microscope.

### 2.20 Cranial defect repair experiment

The experiment was performed as previously described.^19^ Briefly, 8‐week‐old C57 mice were anesthetized by chloral hydrate. An incision was made in the cranial skin. The subcutaneous tissue was carefully removed, and a cranial defect was created using a drill with a 2.5‐mm sterilized drill bit. Then, bone fragments were carefully removed, and 1 × 10^6^ BMSCs were added to the defect. The skin was sutured immediately, and the mouse skull was acquired for micro‐CT analysis after 8 weeks.

### 2.21 Statistical analyses

The results of this study were analyzed by using SPSS 22.0 software. The results are expressed as the mean ± standard deviation (SD). The correlation analysis was performed by Pearson correlation and linear regression analysis. Differences between different groups were analyzed by using independent‐sample *t*‐tests. *p* < 0.05 was regarded as significant.

## 3 RESULTS

### 3.1 LncRNA HHAS1 is upregulated during osteogenesis and correlates with BMSC osteogenic differentiation

**FIGURE 1:**
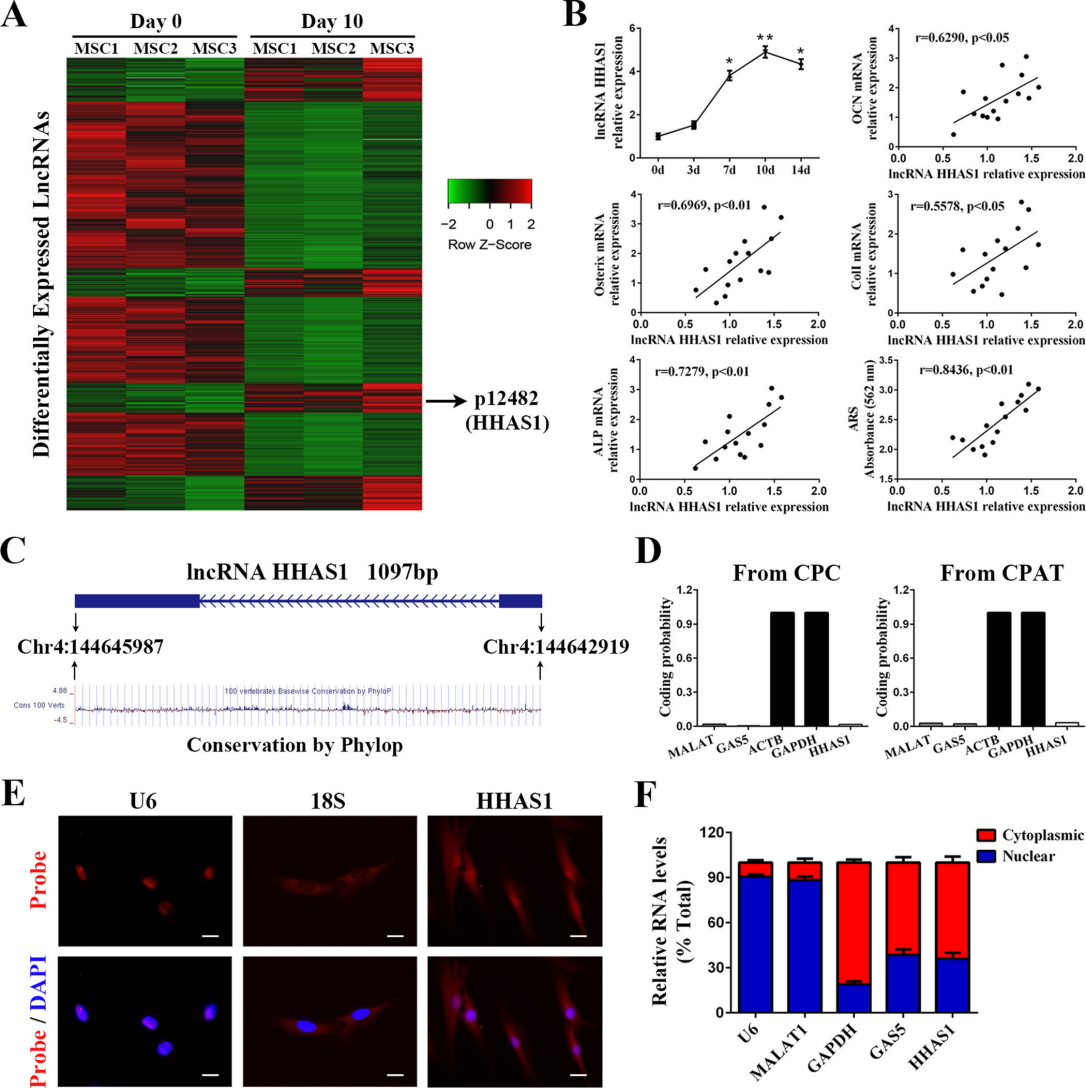
LncRNA HHAS1 is upregulated during the osteogenic differentiation of BMSCs. (A) The heat map of microarray showed the differently expressed lncRNAs of BMSCs 10 days after osteogenic induction. (B) The relative expression levels of lncRNA HHAS1 were upregulated during the osteogenic differentiation of BMSCs and positively correlated with the mRNA expression of OCN, Osterix, ColI, ALP and the intensity of ARS staining. (C) Schematic annotation of the lncRNA HHAS1 genomic locus on chromosome 4 and the conservation analysis of HHAS1 by Phylop on the UCSC browser. The rectangles represent exons. (D) The coding potential was analyzed by CPC and CPAT, which indicated that lncRNA HHAS1 had very low coding potential. (E) The intracellular localization of lncRNA HHAS1 in BMSCs was measured by RNA‐FISH assays. U6 was the nuclear control, and 18S was the cytoplasmic control (scale bar = 10 μm). (F) The percentages of nuclear and cytoplasmic RNA were measured by qPCR after subcellular BMSCs fractionation. U6 and MALAT1 served as nuclear controls, GAPDH served as the cytoplasmic control, and GAS5 served as both a nuclear and cytoplasmic control. The correlation data in (B) were determined by Pearson correlation and linear regression analysis (*n* = 15). Other results are presented as the mean ± SD (*n* = 10, determined by independent‐sample t‐tests). All experiments were performed three independent times, *p < 0.05, **p < 0.01

The characteristics of BMSCs were evaluated and met the international standard for MSC identification (Figures S1A and S1B).^20^ We performed a microarray assay, and the results revealed that the expression profiles of lncRNAs in BMSCs changed dramatically on day 10 after the induction of osteogenic differentiation compared with day 0 (Figure 1A).^8^ Then, we induced the osteogenic differentiation of BMSCs, which was detected by ARS and ALP staining (Figure S2A) and examined the levels of differentially expressed lncRNAs from the microarray. We determined that lncRNA HHIP antisense RNA 1, which we termed HHAS1, was increased significantly during BMSC osteogenesis and reached the highest level on day 10 (Figure 1B). In addition, HHAS1 expression was positively related to the levels of several osteogenesis‐related genes, including OCN, Osterix, ColI, and ALP and the intensity of ARS staining (Figure 1B). These results indicated that HHAS1 might play a crucial role in BMSC osteogenesis; thus, we selected this lncRNA for further investigation.

Subsequently, a RACE assay was performed to obtain the exact sequence of HHAS1 (Figures S3A and S3B) in BMSCs. The location of HHAS1 in the human genome is shown (Figure 1C). In addition, we analyzed the conservation of HHAS1 using the UCSC Genome Browser, and the results showed that HHAS1 was modestly conserved in primates (Figure 1C). Then, we tested the protein‐coding potential of HHAS1 by Coding Potential Calculator (CPC) and Coding Potential Assessment Tool (CPAT), with ACTB and GAPDH, two well‐known protein‐coding genes as protein‐coding controls and with MALAT and GAS5, two classical long non‐coding RNA that have been reported to lack protein‐coding potential as non‐coding controls. Both of these tools revealed that HHAS1 had a very low coding potential (Figure 1D). Furthermore, we determined the cellular localization of HHAS1 by FISH and found that HHAS1 was present in both the nucleus and cytoplasm (Figure 1E), which was verified by nuclear/cytoplasmic fractionation‐qPCR examination (Figure 1F). Overall, we identified a lncRNA, HHAS1, that was upregulated during osteogenesis and correlated with BMSC osteogenic differentiation.

### 3.2 HHAS1 facilitates BMSC osteogenic differentiation

**FIGURE 2:**
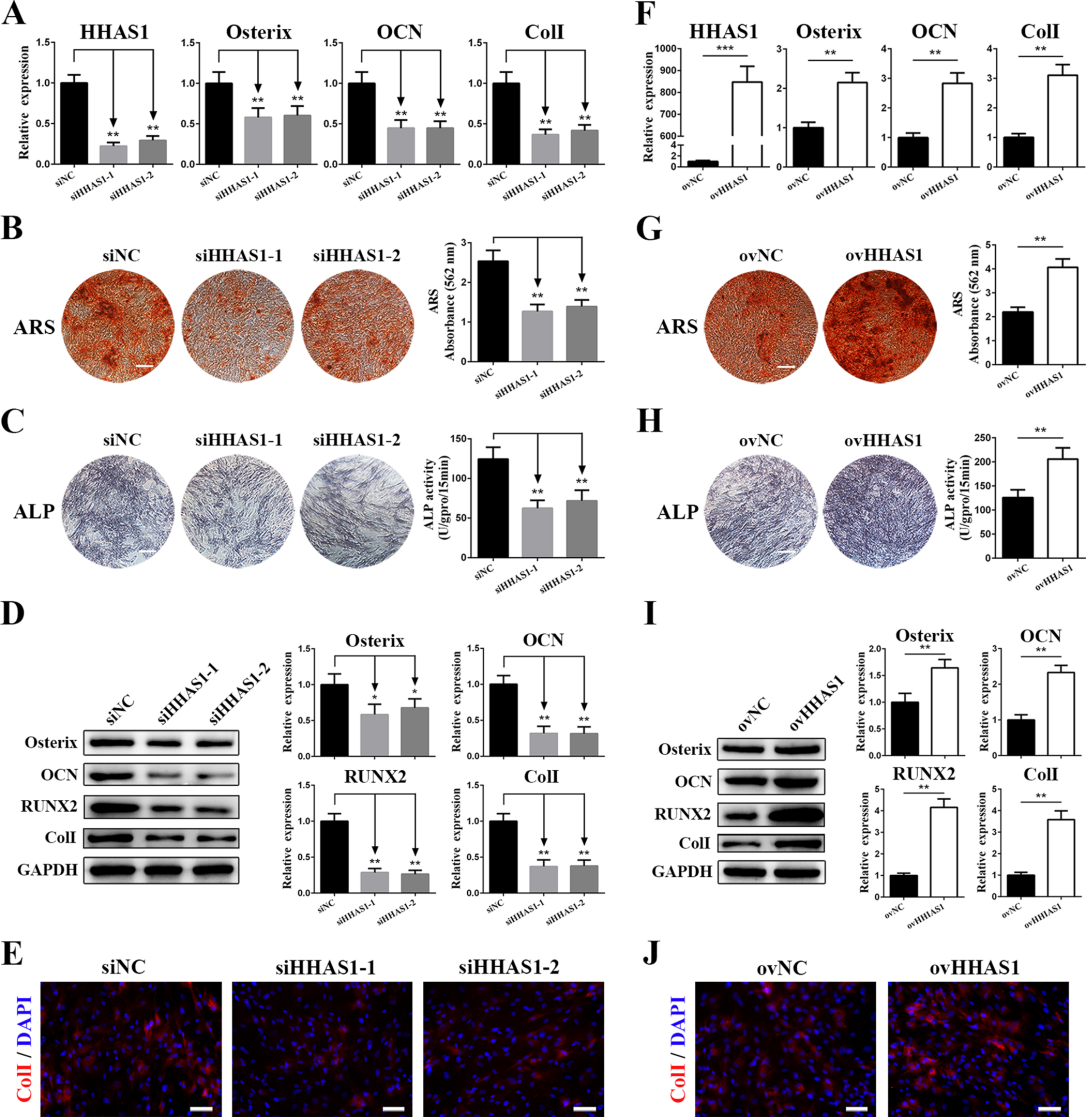
HHAS1 positively regulates the osteogenic differentiation of BMSCs. HHAS1 expression was downregulated by siRNAs or overexpressed by lentivirus. (A) The levels of HHAS1 were decreased by both siRNAs, and the mRNA; levels of Osterix, OCN and ColI were reduced with HHAS1 downregulation. (B) Both siRNAs targeting HHAS1 significantly decreased the ARS staining of BMSCs (scale bar = 200 μm). (C) Both siRNAs targeting HHAS1 significantly decreased the ALP staining and ALP activity of BMSCs (scale bar = 200 μm). (D) Both siRNAs targeting HHAS1 successfully reduced the protein levels of Osterix, OCN, RUNX2, and ColI in BMSCs. (E) Immunofluorescence detection indicated that both siRNAs targeting HHAS1 significantly reduced the ColI signal in BMSCs (scale bar = 50 μm). (F) HHAS1 levels were significantly elevated by the overexpression lentivirus, and the mRNA levels of Osterix, OCN and ColI were increased with HHAS1 overexpression. (G) Overexpressing HHAS1 significantly enhanced the ARS staining of BMSCs (scale bar = 200 μm). (H) Overexpressing HHAS1 markedly increased the ALP staining and ALP activity of BMSCs (scale bar = 200 μm). (I) Overexpressing HHAS1 successfully elevated the protein levels of Osterix, OCN, RUNX2 and ColI in BMSCs. (J) Immunofluorescence detection indicated that overexpressing HHAS1 significantly increased the ColI signal in BMSCs (scale bar = 50 μm). The data are presented as the mean ± SD (*n* = 10, determined by independent‐sample t‐tests). All experiments were performed three independent times, *p < 0.05, **p < 0.01, ***p < 0.001

To explore the effect of HHAS1 on BMSC osteogenesis, we first downregulated HHAS1 expression in BMSCs using siRNAs. Both siRNAs downregulated the levels of HHAS1 and the gene levels of Osterix, OCN and ColI (Figure 2A). In addition, the downregulation of HHAS1 expression impaired the ARS staining intensity and ALP activity (Figures 2B and 2C). Furthermore, the levels of Osterix, OCN, RUNX2, and ColI proteins, as determined by western blotting (Figure 2D), and the ColI signal, measured by immunofluorescence (Figure 2E), were decreased with HHAS1 siRNA treatment. Then, we overexpressed HHAS1 by lentivirus transduction and obtained the opposite results (Figures 2F‐2J). Taken together, these data showed that HHAS1 positively regulated the osteogenic differentiation of BMSCs.

### 3.3 HHAS1 promotes BMSC osteogenic differentiation in vivo

**FIGURE 3:**
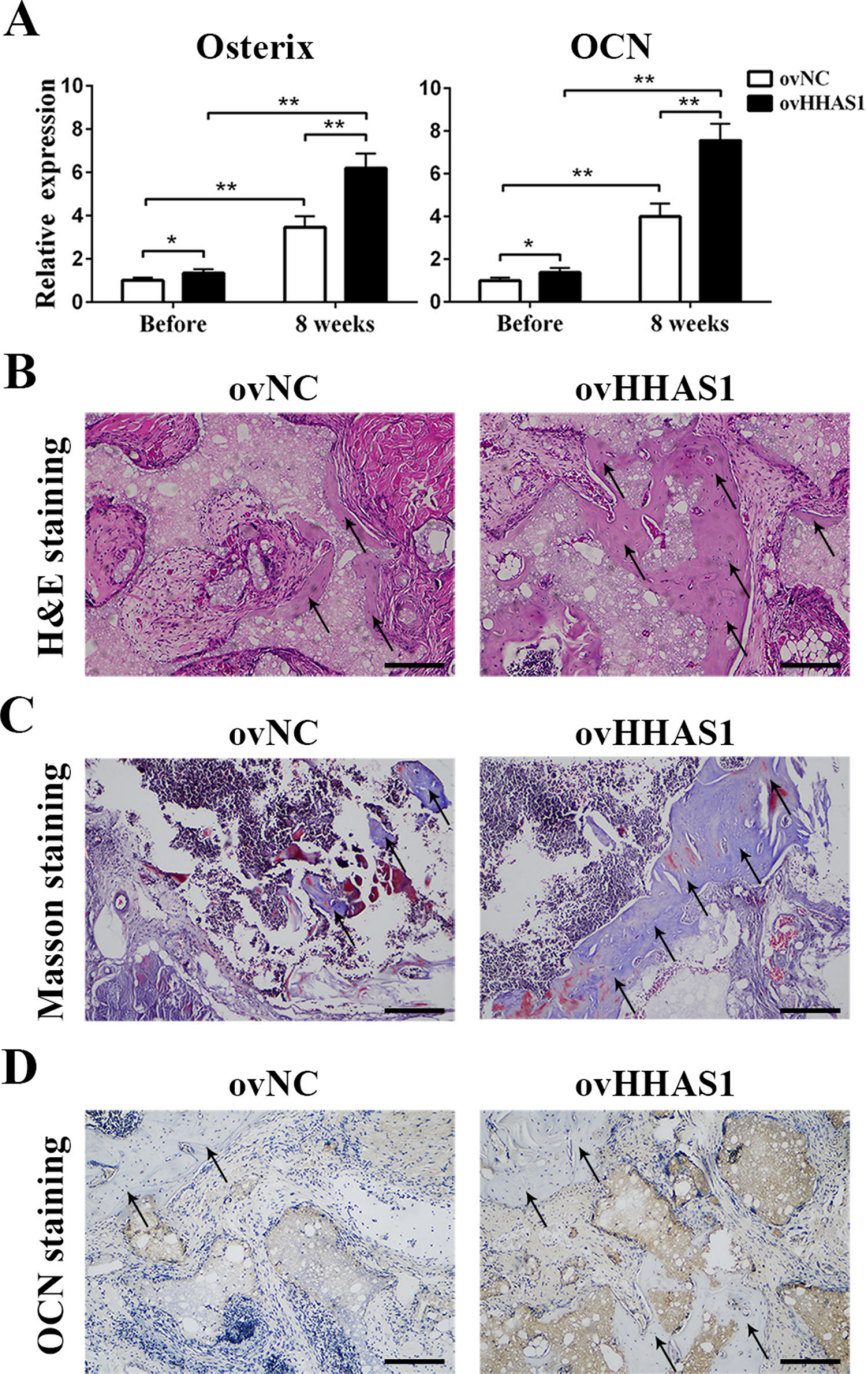
HHAS1 promotes BMSC osteogenic differentiation in vivo. BMSCs were loaded onto scaffolds and transplanted into nude mice. (A) The qPCR results showed that after implantation, the expression of Osterix and OCN increased obviously, and the HHAS1‐overexpression group displayed higher levels of these genes than the control group. (B) H&E staining showed more new bone formation in the HHAS1‐overexpression group (scale bar = 100 μm). (C) Masson's trichrome staining revealed that the HHAS1‐overexpression group had increased collagen organization (scale bar = 100 μm). (D) Immunohistochemistry showed that the HHAS1‐overexpression group displayed stronger staining of OCN (scale bar = 100 μm). The black arrows indicate the area of new bone formation. All experiments were performed three independent times, *n* = 5

To determine the function of HHAS1 in BMSC osteogenesis in vivo, BMSCs were transfected with a negative control vector or an HHAS1‐overexpression lentiviral vector and cultured with osteoblast‐inducing conditional media for 5 days in vitro. Then, the BMSCs were transferred to the scaffolds and transplanted under the dorsal skin of mice. Eight weeks later, the implants were harvested for osteogenesis examination. First, we collected the RNA from the BMSC implants before and 8 weeks after implantation. The qPCR results indicated that the expression of OCN and Osterix was enhanced significantly after implantation, and the HHAS1‐overexpression group displayed higher levels of these genes than the control group (Figure 3A). Furthermore, H&E staining displayed that the HHAS1‐overexpression group displayed more obvious new bone formation than the control group (Figure 3B). Masson's trichrome staining revealed an increase in collagen organization (Figure 3C), and the immunohistochemical staining of OCN showed a stronger staining intensity in HHAS1‐overexpression group (Figure 3D). In summary, these data indicated that HHAS1 could promote BMSC osteogenesis in vivo.

### 3.4 HHAS1 regulates BMSC osteogenesis through serving as a ceRNA to sponge miR‐204‐5p

**FIGURE 4:**
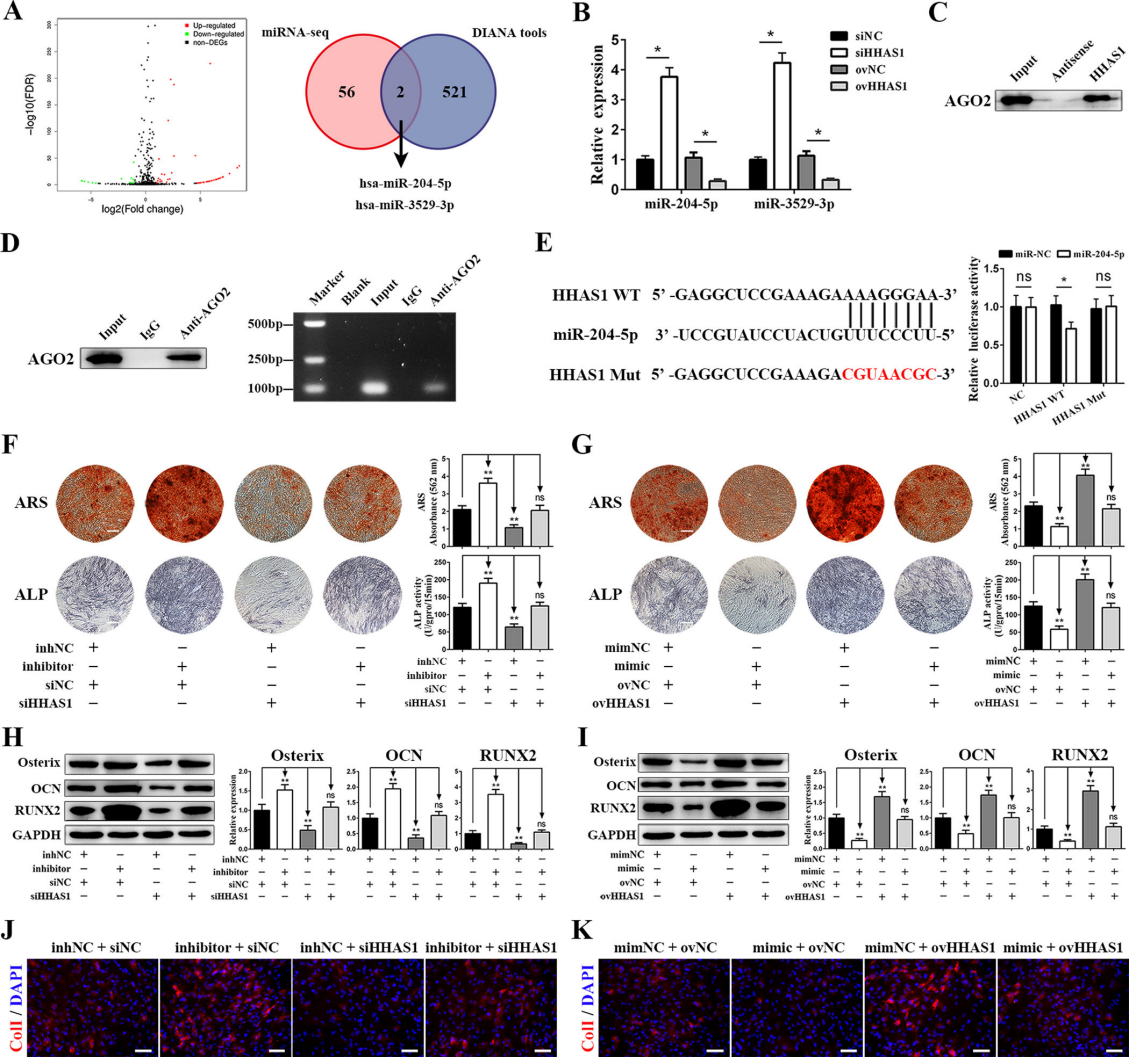
HHAS1 regulates BMSC osteogenesis by acting as a ceRNA to sponge miR‐204‐5p. (A) Volcano plots of miRNA sequencing analysis (left) and the intersection between the results of miRNA sequencing and the prediction analysis by DIANA Tools (right). (B) Silencing HHAS1 increased the levels of miR‐204‐5p and miR‐3529‐3p, and HHAS1 overexpression reduced the levels of these two miRNAs. (C) RNA pull‐down assay showed that HHAS1 interacted with the AGO2 protein. (D) RIP assay showed that anti‐AGO2 antibody successfully precipitated the AGO2 protein (left), and the subsequent PCR electrophoresis assay showed that the AGO2 protein interacted with HHAS1 (right). (E) The binding sites of WT HHAS1 and the mutated sites in MUT HHAS1 (left). Dual‐luciferase reporter assays showed that the miR‐204‐5p mimic inhibited the luciferase activity of the HHAS1 WT group but not the HHAS1 MUT group (right). (F) The miR‐204‐5p inhibitor promoted the ARS staining, ALP staining, and ALP activity of BMSCs and rescued the effect of HHAS1 silencing (scale bar = 200 μm). (G) The miR‐204‐5p mimic decreased the ARS staining, ALP staining, and ALP activity of BMSCs and rescued the effect of HHAS1 overexpression (scale bar = 200 μm). (H) The miR‐204‐5p inhibitor increased the protein levels of Osterix, OCN, and RUNX2 in BMSCs and rescued the effect of HHAS1 silencing. (I) The miR‐204‐5p mimic decreased the protein levels of Osterix, OCN, and RUNX2 in BMSCs and rescued the effect of HHAS1 overexpression. (J) The miR‐204‐5p inhibitor enhanced the fluorescence signal of ColI and rescued the effect of HHAS1 silencing (scale bar = 50 μm). (K) The miR‐204‐5p mimic decreased the fluorescence signal of ColI and rescued the effect of HHAS1 overexpression (scale bar = 50 μm). The data in B and E to I are presented as the mean ± SD (*n* = 10, determined by independent‐sample *t*‐tests). All experiments were performed three independent times, ns = not statistically significant, *p < 0.05, **p < 0.01

Considering that more HHAS1 is located in the cytoplasm and that cytoplasm‐located lncRNAs usually function by interacting with miRNAs,^21^ we performed a small RNA sequencing assay, which revealed that 58 miRNAs were upregulated when HHAS1 was downregulated (Figure 4A). In addition, we used DIANA Tools to predict miRNAs that potentially interacted with HHAS1. The intersection of the results of miRNA sequencing and the prediction analysis identified two miRNAs, miR‐204‐5p, and miR‐3529‐3p (Figure 4A). Then, we measured the function of HHAS1 on the level of these two miRNAs by qPCR. Downregulating HHAS1 expression enhanced the levels of miR‐204‐5p and miR‐3529‐3p, and conversely, overexpressing HHAS1 reduced the levels of these miRNAs (Figure 4B). Additionally, we investigated whether HHAS1 could interact with AGO2, a crucial protein of the RNA‐induced silencing complex,^22^ and the results of RNA pull‐down and RIP assays jointly indicated a mutual interaction of HHAS1 and AGO2 (Figures 4C and 4D). Furthermore, the results of dual‐luciferase reporter assay indicated that the luciferase activity of the HHAS1 WT construct, but not the MUT construct or the negative control vector, was impaired by the mimics of both miR‐204‐5p (Figure 4E) and miR‐3529‐3p (Figure S4A).

Next, we explored the effect of these two miRNAs on BMSC osteogenesis. The results indicated that an inhibitor of miR‐204‐5p enhanced ARS staining, ALP activity, the expression of Osterix, OCN, and Runx2 and the fluorescence signal of ColI and successfully reversed the effect of HHAS1 silencing (Figures 4F, 4H, and 4J). Conversely, the miR‐204‐5p mimic exerted the reverse effect and rescued the effect of HHAS1 overexpression (Figures 4G, 4I, and K). However, miR‐3529‐3p did not impact BMSC osteogenesis (Figures S4B and S4C). Overall, by acting as a ceRNA, HHAS1 sponged miR‐204‐5p thus regulating BMSC osteogenesis.

### 3.5 RUNX2 is the downstream gene responsible for BMSC osteogenesis regulation

**FIGURE 5:**
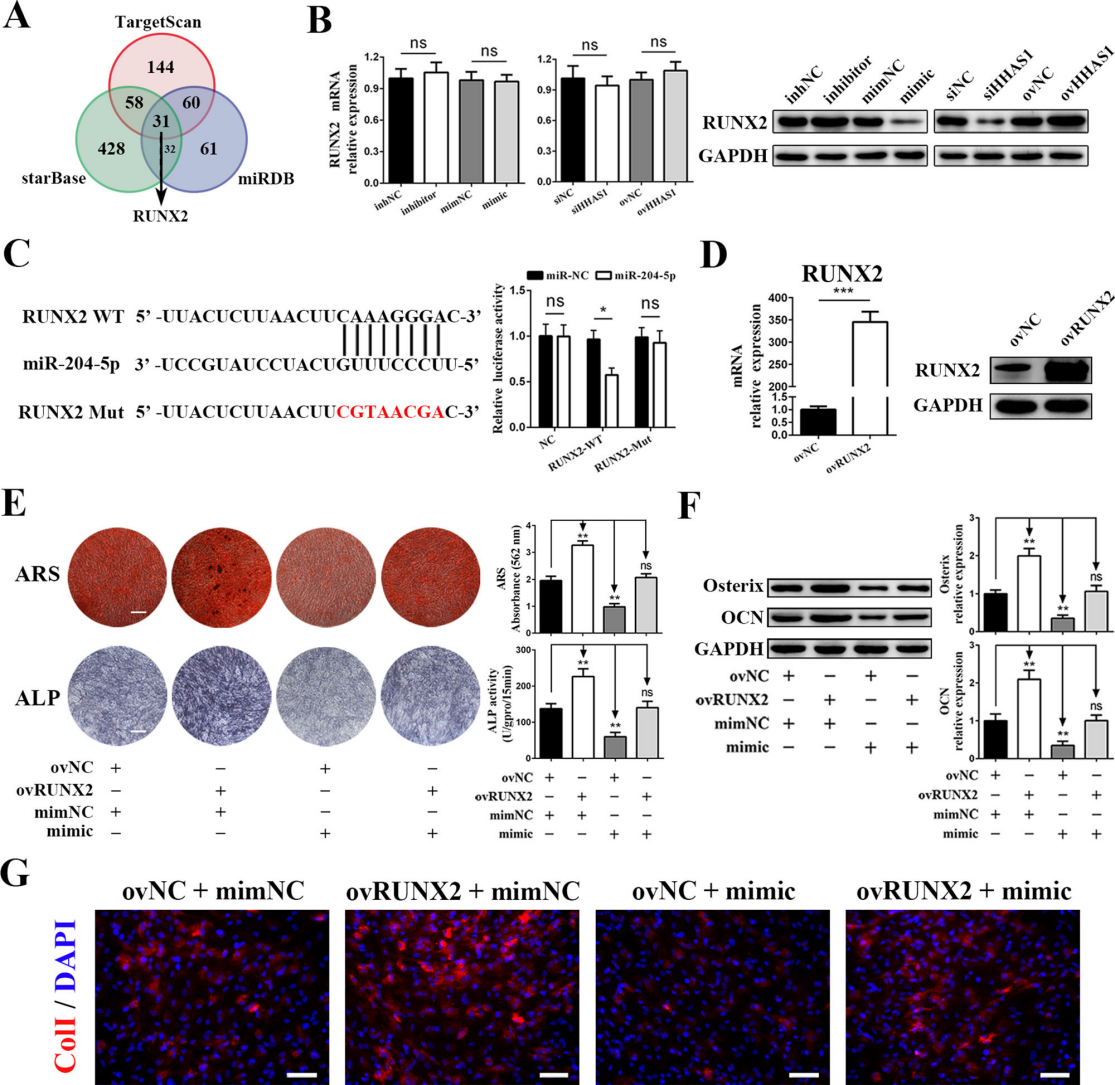
RUNX2 is the target gene of miR‐204‐5p responsible for regulating BMSC osteogenesis. (A) The prediction results of the potential target genes of miR‐205‐5p from TargetScan, starBase, and miRDB database. (B) The miR‐204‐5p mimic, miR‐204‐5p inhibitor, HHAS1 siRNA, and HHAS1‐overexpressing lentivirus did not influence the mRNA levels (left) but changed the protein levels of RUNX2 (right). (C) The binding sites of WT RUNX2 and mutated sites in MUT RUNX2 (left). Dual‐luciferase reporter assays showed that the miR‐204‐5p mimic inhibited the luciferase activity of the RUNX2 WT group but not the RUNX2 MUT group (right). (D) The efficiency of the RUNX2 overexpression was measured by qPCR (left) and western blotting (right). (E) Overexpressing RUNX2 significantly increased the ARS staining, ALP staining and ALP activity of BMSCs and reversed the effect of miR‐204‐5p mimic (scale bar = 200 μm). (F) Overexpressing RUNX2 markedly increased the protein levels of Osterix and OCN in BMSCs and reversed the effect of miR‐204‐5p mimic. (G) Overexpressing RUNX2 significantly increased the fluorescence signal of ColI in BMSCs and reversed the effect of miR‐204‐5p mimic (scale bar = 50 μm). The data are presented as the mean ± SD (n = 10, determined by independent‐sample *t*‐tests). All experiments were performed three independent times, ns = not statistically significant, *p < 0.05, **p < 0.01, ***P < 0.001

To identify the target genes of miR‐204‐5p, we performed prediction analysis of potential target genes using the TargetScan, starBase and miRDB databases. The prediction results showed 293, 549, and 183 potential genes in TargetScan (cumulative weighted context++ score ≤ ‐0.2), starBase (high stringency [≥3]) and miRDB databases (Target score ≥ 90), respectively. Among the intersection of the three prediction results, we found a gene highly related to osteogenic differentiation—RUNX2 (Figure 5A). Then, we explored whether miR‐204‐5p regulated the level of RUNX, and we found that the miR‐204‐5p inhibitor enhanced, while the miR‐204‐5p mimic reduced, the protein level of RUNX2 but not the mRNA level (Figure 5B). In addition, HHAS1 did not affect the mRNA expression of RUNX2, but silencing HHAS1 reduced, while overexpressing HHAS1 enhanced, the protein levels of RUNX2 (Figure 5B). Then, we constructed dual‐luciferase reporters containing RUNX2 WT or RUNX2 MUT, which was mutated according to the prediction of binding site. We found that the mimic decreased the luciferase activity of the RUNX2 WT construct and had no significant effect on the MUT reporter construct or the negative control vector (Figure 5C).

Subsequently, we downregulated RUNX2 expression by siRNAs (Figure S5A) and induced BMSC osteogenesis. The results showed that interfering with RUNX2 significantly decreased the ColI fluorescence signal (Figure S5B), ARS staining, ALP activity (Figure S5C) and the levels of Osterix and OCN (Figure S5D). Furthermore, we overexpressed RUNX2 via lentivirus, and the results showed that RUNX2 expression was successfully enhanced (Figure 5D). Then, we detected the effect of RUNX2 overexpression on BMSC osteogenesis. The results showed that RUNX2 overexpression enhanced ARS staining, ALP activity and the levels of Osterix, OCN and ColI and successfully rescued the function of miR‐204‐5p mimic (Figures 5E–5G). In summary, these findings revealed that RUNX2 was the downstream gene of miR‐204‐5p involved in BMSC osteogenesis.

### 3.6 IRF2 promotes the transcription of HHAS1 during BMSC osteogenic differentiation

**FIGURE 6:**
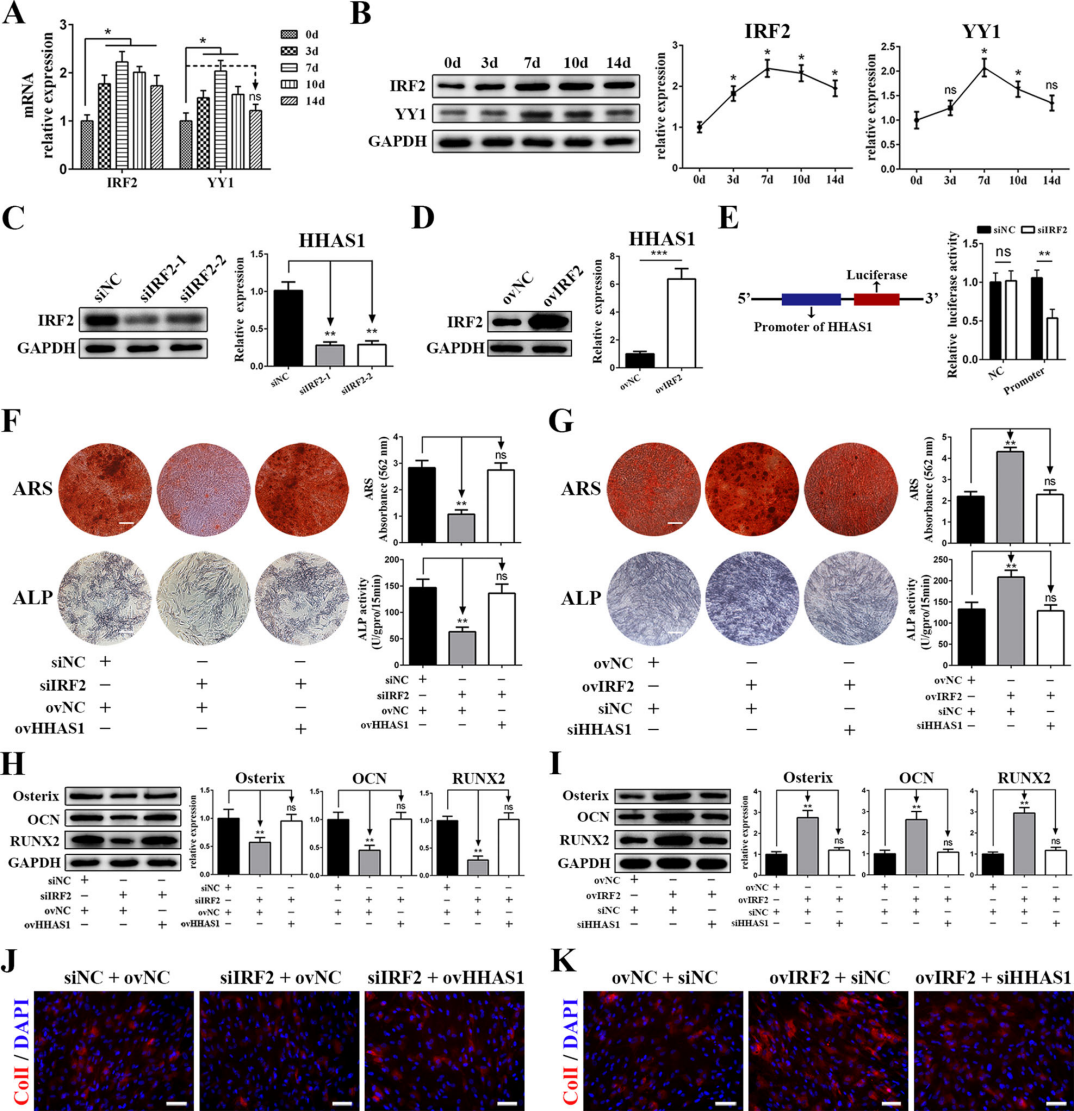
IRF2 promotes the transcription of HHAS1 during BMSC osteogenic differentiation. (A) The mRNA levels of predicted transcription factor, IRF2, and YY1 in BMSCs during osteogenic differentiation. The levels of IRF2 and YY1 increased and reached a maximum on day 7. (B) The protein levels of IRF2 and YY1 in BMSCs during osteogenic differentiation. (C) The efficiency of siRNAs targeting IRF2 was measured by western blotting (left), and HHAS1 expression was decreased by IRF2 siRNAs (right). (D) The efficiency of IRF2 overexpression was measured by western blotting (left), and HHAS1 expression was increased by IRF2 overexpression (right). (E) The HHAS1 promoter was inserted into the 5′ end of the luciferase gene. Silencing IRF2 significantly inhibited the luciferase activity of the promoter group. (F) Silencing IRF2 markedly decreased the ARS staining, ALP staining and ALP activity of BMSCs, while overexpressing HHAS1 abrogated these effects (scale bar = 200 μm). (G) Overexpressing IRF2 significantly enhanced the ARS staining, ALP staining and ALP activity of BMSCs, while silencing HHAS1 abrogated these effects (scale bar = 200 μm). (H) Silencing IRF2 significantly reduced the protein levels of Osterix, OCN, and RUNX2 in BMSCs, while overexpressing HHAS1 abrogated these effects. (I) Overexpressing IRF2 significantly enhanced the protein levels of Osterix, OCN, and RUNX2 in BMSCs, while silencing HHAS1 abrogated these effects. (J) Silencing IRF2 significantly impaired the fluorescence signal of ColI in BMSCs, while overexpressing HHAS1 abrogated these effects (scale bar = 50 μm). (K) Overexpressing IRF2 significantly strengthened the fluorescence signal of ColI in BMSCs, while silencing HHAS1 abrogated these effects (scale bar = 50 μm). The data are presented as the mean ± SD (*n* = 10, determined by independent‐sample *t*‐tests). All experiments were performed three independent times, ns = not statistically significant, *p < 0.05, **p < 0.01, ***p < 0.001

To determine the mechanism by which HHAS1 expression was increased during BMSC osteogenic differentiation, we predicted the TFs potentially binding to the promoter of HHAS1 using PROMO. The prediction results revealed 10 of TFs, including ATF2, FOXP3, IRF2, XBP1, YY1, P53, STAT4, Pax5, C/EBP beta, c‐Myc, TFIID, HNF1A, and WT1. We examined the expression of these TFs, and found that the levels of IRF2 and YY1 but not the others were increased during BMSC osteogenic differentiation (Figures 6A, 6B, and S6A). Then, we downregulated the levels of these two factors by siRNAs (Figures 6C and S6B). We found that the HHAS1 level was reduced by knockdown of IRF2 (Figure 6C) but not YY1 (Figure S6C), while the overexpression of IRF2 had the opposite effect (Figure 6D). We further constructed a dual‐luciferase reporter construct containing the promoter of HHAS1 and found that downregulating IRF2 inhibited the luciferase activity of the promoter‐containing construct but not the negative control vector (Figure 6E). These results revealed that the TF IRF2 activated the promoter of HHAS1, thus facilitating HHAS1 transcription during BMSC osteogenic differentiation.

Next, we investigated the role of IRF2 in BMSC osteogenesis, and we found that the knockdown of IRF2 significantly inhibited the ARS staining intensity (Figure 6F), ALP activity (Figure 6F), the protein levels of Osterix, OCN and RUNX2 (Figure 6H) and the fluorescence signal of ColI (Figure 6J) in BMSCs. In addition, these effects of IRF2 were abrogated by the overexpression of HHAS1 (Figures 6F, 6H, and 6J). Furthermore, we overexpressed IRF2 and obtained the opposite results, and the effect of IRF2 overexpression on BMSC osteogenesis was rescued by HHAS1 knockdown (Figures 6G, 6I, and 6K). Taken together, these findings showed that IRF2 was the upstream modulator of HHAS1 in regulating BMSC osteogenic differentiation.

### 3.7 HHAS1 and IRF2 improve bone defect repair in vivo

**FIGURE 7:**
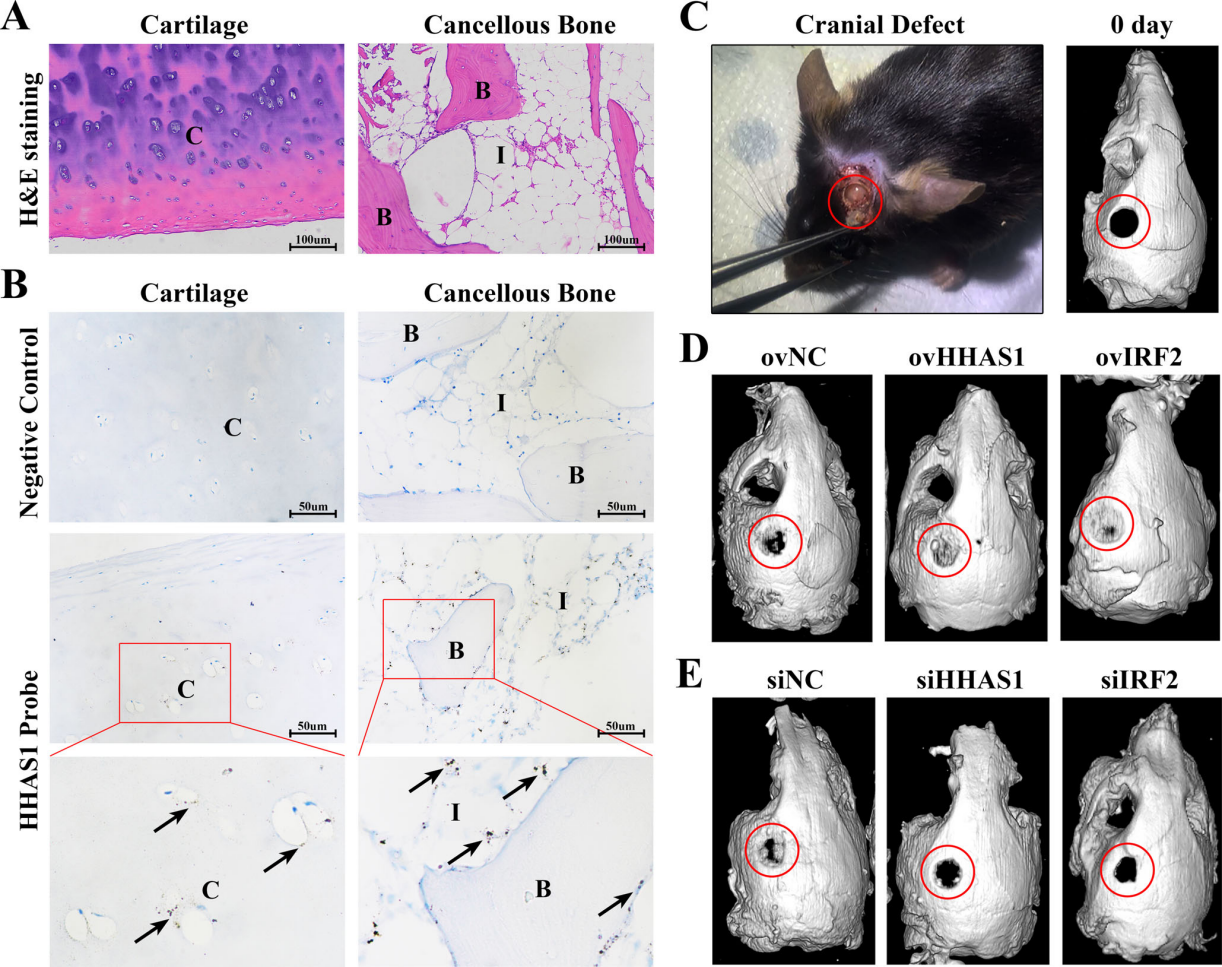
HHAS1 and IRF2 help improve bone defect repair in vivo. (A) The H&E staining results showed the histomorphology of cartilage and cancellous bone. (B) RNAscope assay was performed on human femoral head specimens. The negative control group did not show any brown dots, while the HHAS1 probe group showed brown dots on both cartilage area and cancellous bone area, indicating the expression of HHAS1. *n* = 3. (C) The cranial defect performed on mice (left) and the micro‐CT scans of mouse skulls 0 days after cranial defect establishment (right). (D) The results of micro‐CT showed that HHAS1‐overexpressing group and IRF2‐overexpressing group displayed improved bone repair than control group. (E) The results of micro‐CT showed that HHAS1‐silencing group and IRF2‐silencing group displayed impaired bone repair compared with the control group. *n* = 5. C indicates cartilage. B indicates bone. I indicates interstitial cells. The black arrow indicates HHAS1. The red circle indicates bone defect area

To detect whether HHAS1 exists in bone tissue, we performed an RNAscope in situ hybridization assay, a specific and sensitive method to detect RNA in cells and tissues with morphological context,^23^ on a human femur head. The H&E staining results showed the histomorphology of the specimens (Figure 7A). The results of RNAscope staining indicated that HHAS1 was expressed in both the cartilage area and cancellous bone area, especially in the interstitial cells of the cancellous bone area (Figure 7B). To further evaluate the effect of HHAS1 and IRF2 on osteogenesis in vivo, we constructed mouse cranial defect models (Figure 7C). The results showed that compared with control BMSCs, both HHAS1‐overexpressing BMSCs and IRF2‐overexpressing BMSCs improved the mouse cranial defect to a greater extent (Figure 7D), while HHAS1‐silencing BMSCs and IRF2‐silencing BMSCs displayed the opposite effect (Figure 7E). These results indicated that HHAS1 and IRF2 could help improve bone defect repair in vivo.

## 4 DISCUSSION

**FIGURE 8:**
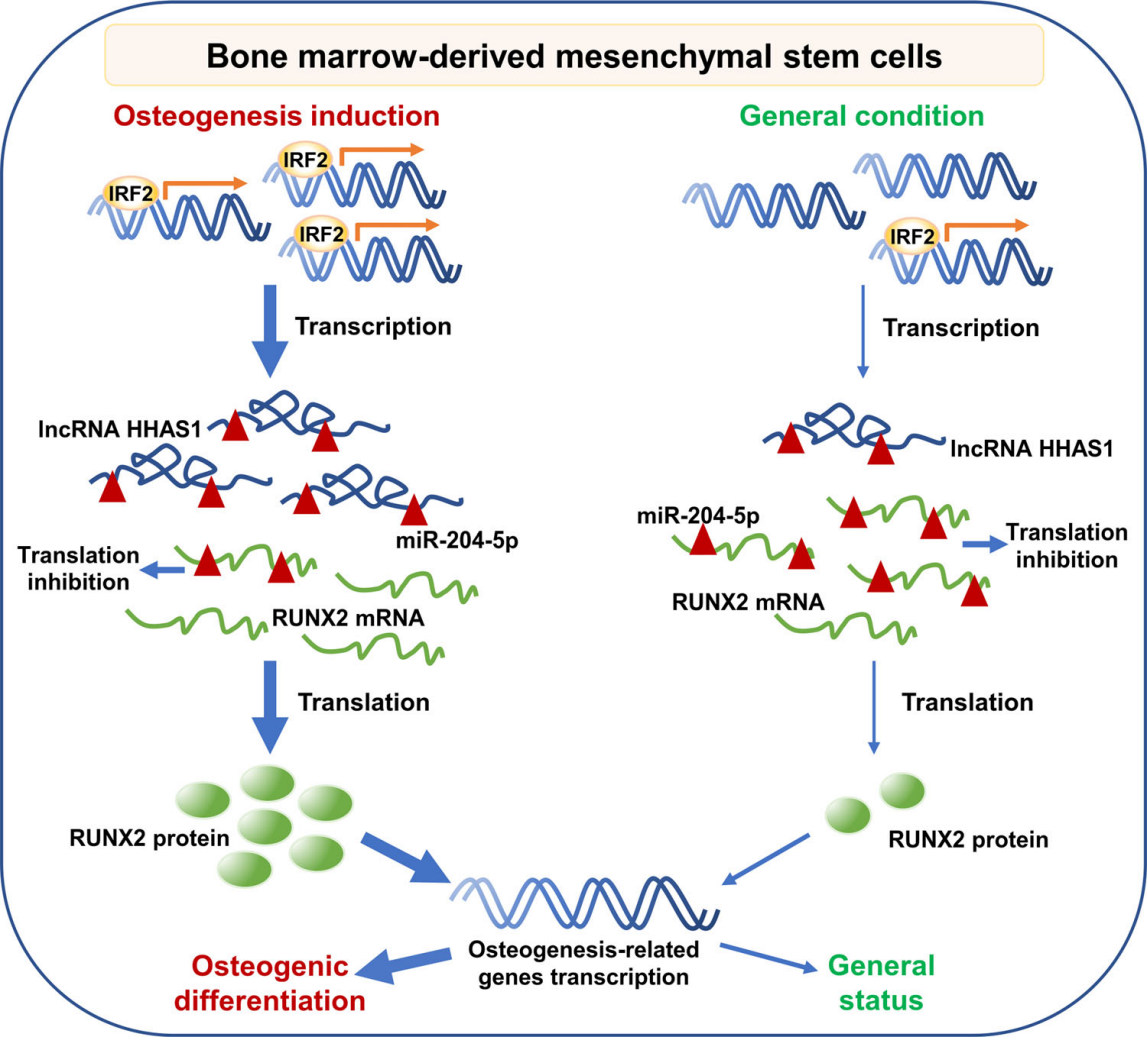
A schematic presentation of IRF2/HHAS1/miR‐204‐5p/RUNX2 axis on BMSC osteogenic differentiation. Under osteogenic induction, increased IRF2 promotes the transcription of HHAS1. Subsequently, HHAS1 sponges miR‐204‐5p and protects RUNX2 mRNA from translation inhibition, therefore mediating enhanced RUNX2 protein production and accelerating osteogenic differentiation

In this study, we newly annotated a lncRNA, HHAS1, that was increased during BMSC osteogenesis and facilitated the osteogenic differentiation of BMSCs. Mechanistically, HHAS1 sponged miR‐204‐5p by acting as a ceRNA and increased the level of RUNX2. In addition, the enhanced level of IRF2 increased its presence on the promoter of HHAS1 and therefore activated the transcription of HHAS1 during BMSC osteogenesis (Figure 8). Furthermore, we found that HHAS1 was expressed in human bone tissue, and IRF2 and HHAS1 helped improve bone defect repair in vivo.

Osteogenesis is an important part of bone metabolism and is involved in many physiological processes and pathologic conditions.^6^ MSCs are the major source of osteoblasts and are of great importance to osteogenesis.^24^ Aberrant osteogenesis of MSCs participates in many diseases, such as osteoporosis^25^, ^26^ and ankylosing spondylitis.^18^ Adjusting the osteogenic differentiation ability of MSCs may help improve some conditions, such as bone mass loss and fracture healing.^27^ Therefore, it is significant to illustrate the regulatory networks of MSC osteogenesis and provide potential targets for osteogenic modification.

Previous researches have reported that lncRNAs are of great important in regulating the biological functions of stem cells, especially stem cell differentiation.^28^, ^29^ Zhou et al^30^ revealed that lncRNA H19 is a crucial factor for the development of embryonic hematopoietic stem cells. Huang et al^31^ showed that lncRNA ADAMTS9‐AS2 controls human MSC chondrogenic differentiation and could be regarded as a therapeutic target for cartilage repair. Thus, it is significant to explore the function of lncRNAs in MSC osteogenesis. We newly annotated a lncRNA, HHAS1, that increased during BMSC osteogenesis and facilitated BMSC osteogenic differentiation. In detail, HHAS1 strengthened ARS and ALP staining, and the levels of Osterix, OCN, RUNX2, and ColI. Of note, mineral is mainly deposited between the collagen fibrils, and ColI constitutes approximately 90% of the bone protein of organic matrix.^32^ Thus, ColI is a very important element to the scaffold upon which mineral is deposited and contributes to bone mechanical properties. In addition, ColI plays a crucial role in bone‐related diseases, including osteoporosis and osteogenesis imperfecta.^33^ We found that HHAS1 promoted BMSC osteogenesis with elevated ColI expression, indicating that HHAS1 played a crucial role in bone formation and might be a treatment target for ColI‐related diseases.

Similar to HHAS1, several lncRNAs, such as HOTAIR, MEG3, H19, and LncRNA ODIR1, are involved in regulating MSC osteogenesis,^7^, ^34^ demonstrating the vital role of lncRNAs in MSC osteogenesis. Unlike the lncRNAs mentioned above, HHAS1 was identified from the lncRNA profile analysis during BMSC osteogenesis that we conducted previously; thus, it may better represent the physiological features of BMSC osteogenesis. In addition, it has been reported that lncRNAs can be located in the cytoplasm or nucleus and can function through a variety of mechanisms.^4^ Several osteogenesis‐related lncRNAs have been reported to have different cellular localizations and molecular mechanisms. Li et al^35^ reported that the cytoplasmic lncRNA H19 promotes the osteogenesis of stem cells through the miR‐141/SPAG9 pathway. Zhu et al^36^ showed that the lncRNA HoxA‐AS3 localizes in the nucleus and suppresses MSC osteogenic differentiation through transcriptional activation. Cao et al^37^ showed that Linc02349 localizes in both the cytoplasm and nucleus and promotes MSC osteogenesis by acting as a ceRNA. We found that HHAS1 was present in both the cytoplasm and the nucleus. Additionally, HHAS1 interacted with AGO2 and miR‐204‐5p, thus facilitating BMSC osteogenic differentiation, a mechanism of cytoplasmic lncRNAs.^21^ However, whether and how nuclear HHAS1 affects BMSCs is still unclear. Commonly, nuclear lncRNAs can execute their regulatory roles in cis by modulating adjacent gene expression or in trans through interacting with splicing factors, regulating the chromatin status, silencing chromosomes or modulating the paraspeckles function.^38^ In addition, we evaluated whether HHAS1 regulated the expression of adjacent genes but obtained negative results (data not provided). Because miR‐204‐5p could completely rescue the effect of HHAS1, we concluded that HHAS1 regulates BMSC osteogenesis mainly through acting as a ceRNA. Further study is required to reveal the function and mechanism of nuclear HHAS1.

MiRNAs play an important role in biological processes, and lncRNAs usually exert their biological functions through interactions with miRNAs.^21^, ^39^ LncRNAs can act as miRNA sponges, therefore repressing the targeting effect of miRNAs on mRNAs, competing with miRNAs for the degradation of target mRNAs, or giving rise to miRNAs.^40^ In our study, we showed that HHAS1 could regulate miRNAs in a typical ceRNA manner. Specifically, HHAS1 was found to share a complementary sequence with miR‐204‐5p and could reduce its expression (Figures 4B and 4E). Additionally, HHAS1 could bind to miR‐204‐5p (Figure 4E) and interact with AGO2 (Figures 4C and 4D), the catalytic component of RISC, further protecting RUNX2 mRNA from miRNA‐mediated translational inhibition (Figures 5A and 5B). In agreement with previous studies, cytoplasmic lncRNAs, such as H19, MALAT1, and lncRNA‐ANCR, usually function by interacting with miRNAs, especially by acting as ceRNAs.^22^

Previously, studies have shown that miRNAs are crucial regulators of bone metabolism and numerous miRNAs play an important role in the regulation of MSC osteogenesis.^10^, ^11^, ^41^ Lv et al^42^ showed that miR‐193a‐3p suppressed the osteogenic differentiation of BMSCs via the MAP3k3 signaling axis, and Li et al^43^ revealed that miR‐216a rescued the suppressive effect of dexamethasone on osteogenesis through the PI3K/AKT pathway. We found that miR‐204‐5p played an inhibitory role in BMSC osteogenesis. Similarly, it has been reported that miR‐204‐5p suppressed osteogenesis. Notably, the cell types were different between our studies, as were the downstream regulatory mechanisms. The study by Xiao et al^14^ revealed that in human aortic valve interstitial cells, miR‐204‐5p inhibited osteogenesis by regulating SMAD4, and the study by Shang et al^44^ demonstrated that miR‐204‐5p inhibited rat BMSC osteogenesis by downregulating RUNX2 expression. Together, the results of these studies and ours indicate that miR‐204‐5p plays an extensive and vital role in bone formation and may be a valuable target for clinical application.

Commonly, miRNAs play a regulatory role in gene expression via inhibiting mRNA translation or accelerating mRNA degradation. In cases of complete pairing with mRNAs, miRNAs can accelerate mRNA degradation and thus downregulate mRNA levels, while with partial pairing with mRNAs, miRNAs usually inhibit mRNA translation but do not influence mRNA degradation.^9^ Our data revealed that miR‐204‐5p partially paired with RUNX2 mRNA and that the mimic and inhibitor did not influence the mRNA levels but changed the protein levels of RUNX2, which are in agreement with previous description of miRNA functions.

TFs are key cellular components of the regulatory network orchestrating gene expression programs that elicit diverse biological responses.^16^ Previously, it was reported that multiple TFs, including CBFA‐1, YAP, and HOXB7, are involved in MSC differentiation.^45^ IRF2, which belongs to the interferon regulatory factor family of TFs, is mainly involved in immune responses and cancer processes.^17^ Whether IRF2 participates in stem cell differentiation has rarely been studied. We are the first to reveal that IRF2 expression increases during BMSC osteogenic differentiation and mediates osteogenesis by amplifying the transcriptional activity of HHAS1. Recently, it was reported that IRF2 participated in modulating the cell fate of some stem cell types,^46^, ^47^ which also indicates an important role for IRF2 in stem cell regulation and requires further exploration.

## 5 CONCLUSIONS

In conclusion, we identified a novel lncRNA, HHAS1, capable of facilitating BMSC osteogenic differentiation in vitro and in vivo, and we are the first to propose an IRF2/HHAS1/miR‐204‐5p/RUNX2 axis in BMSC osteogenesis. These findings help to elucidate the regulatory network of BMSC osteogenesis and provide potential targets for clinical application. However, there are some limitations to our study. Specifically, it remains unclear whether HHAS1 participates in the development of diseases related to bone metabolism and whether targeting the IRF2/HHAS1/miR‐204‐5p/RUNX2 axis is effective in clinical applications. These issues require further investigation.

## CONFLICT OF INTEREST

The authors have no conflict of interest to declare.

## ETHICS APPROVAL AND CONSENT TO PARTICIPATE

This study was approved by the ethics committee of The Eighth Affiliated Hospital, Sun Yat‐sen University (Shenzhen, China). Written informed consent was obtained from all participants. The animal experiments were approved by the Animal Ethical and Welfare Committee of The Eighth Affiliated Hospital, Sun Yat‐sen University.

## CONSENT FOR PUBLICATION

The authors agree with the publication of all the data involved in this article. No data from other entities were used in this study.

## AUTHORS CONTRIBUTIONS

Huiyong Shen, Guiwen Ye, and Yanfeng Wu were responsible for the study conception. Guiwen Ye, Peng Wang, and Zhongyu Xie designed the experiments. Guiwen Ye, Peng Wang, Jinteng Li, Guan Zheng, Wenjie Liu, and Qian Cao performed the experiments. Ming Li, Shuizhong Cen, and Zhaofeng Li conducted data analysis. Wenhui Yu and Guiwen Ye wrote the manuscript. Huiyong Shen, Wenhui Yu, and Zhongyu Xie provided the revision.

## Supporting information

Supporting information

Supporting information

Supporting information

Supporting information

Supporting information

Supporting information

Supporting information

Supporting information

## Data Availability

The data that support the findings of this study are available from the corresponding author on request.
