# Supplementary figures and images for: IRF2‐mediated upregulation of lncRNA HHAS1 facilitates the osteogenic differentiation of bone marrow‐derived mesenchymal stem cells by acting as a competing endogenous RNA
Source: Clin Transl Med. 2021 Jun 20;11(6):e429. doi: 10.1002/ctm2.429 (PMC8214856; doi:10.1002/ctm2.429)

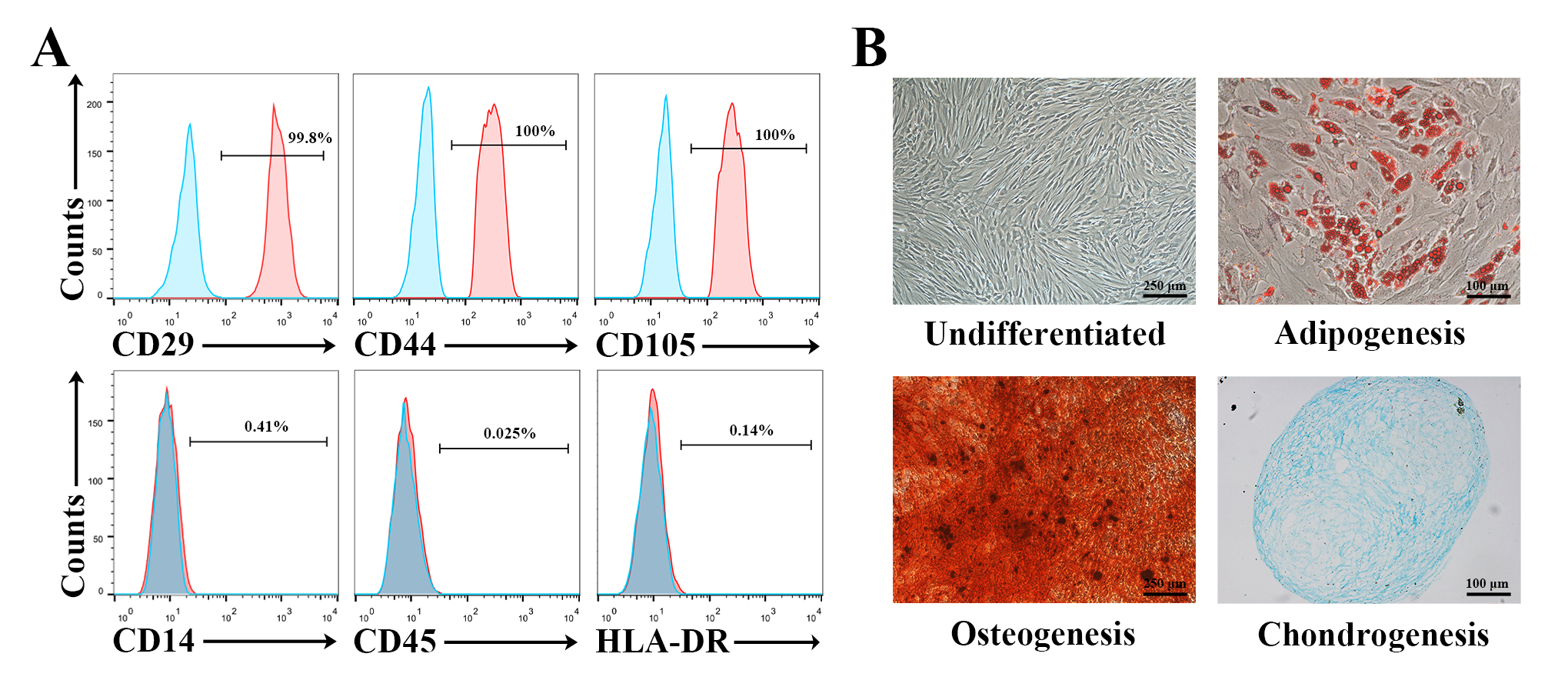

Supplement: Supplementary file 1 — Supporting information [file CTM2-11-e429-s008.tif]

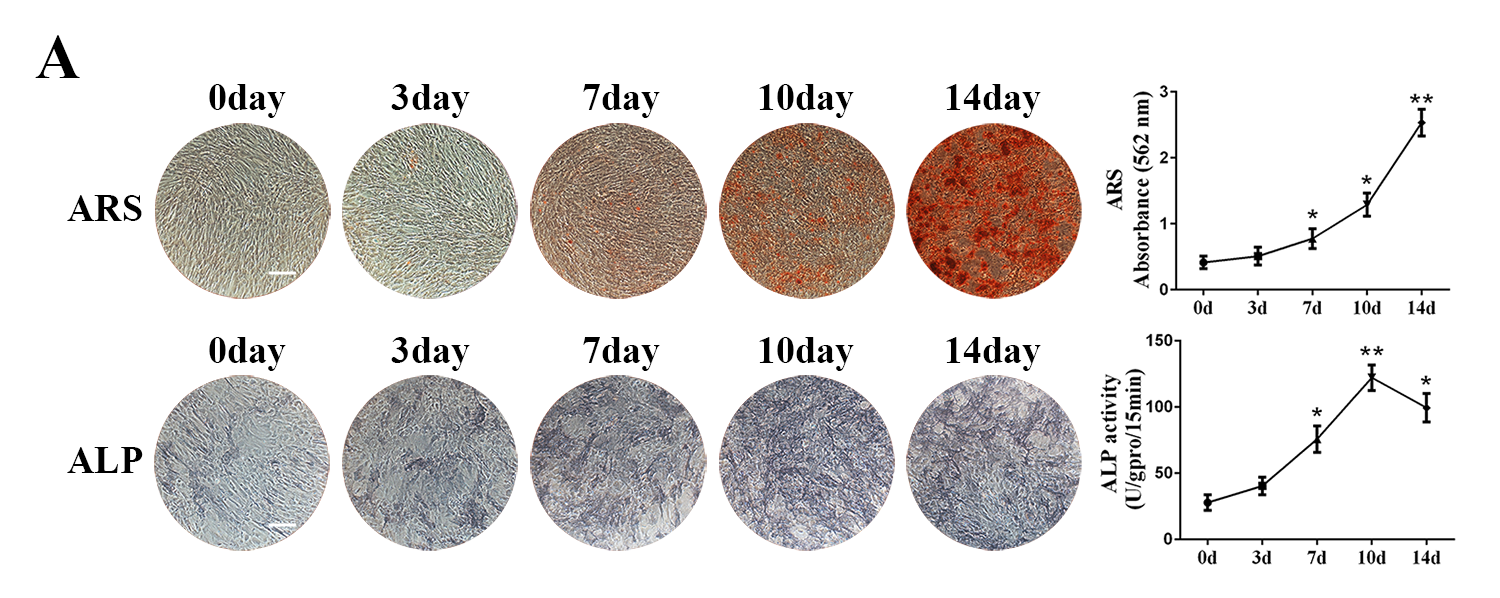

Supplement: Supplementary file 2 — Supporting information [file CTM2-11-e429-s004.tif]

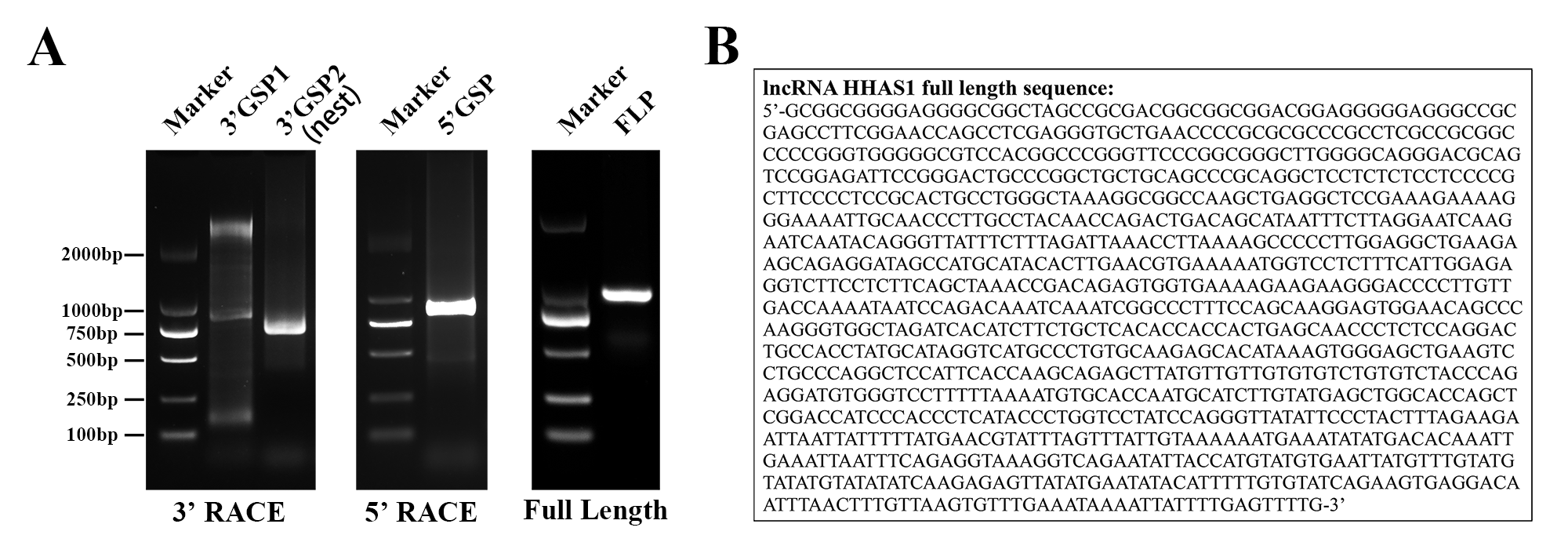

Supplement: Supplementary file 3 — Supporting information [file CTM2-11-e429-s002.tif]

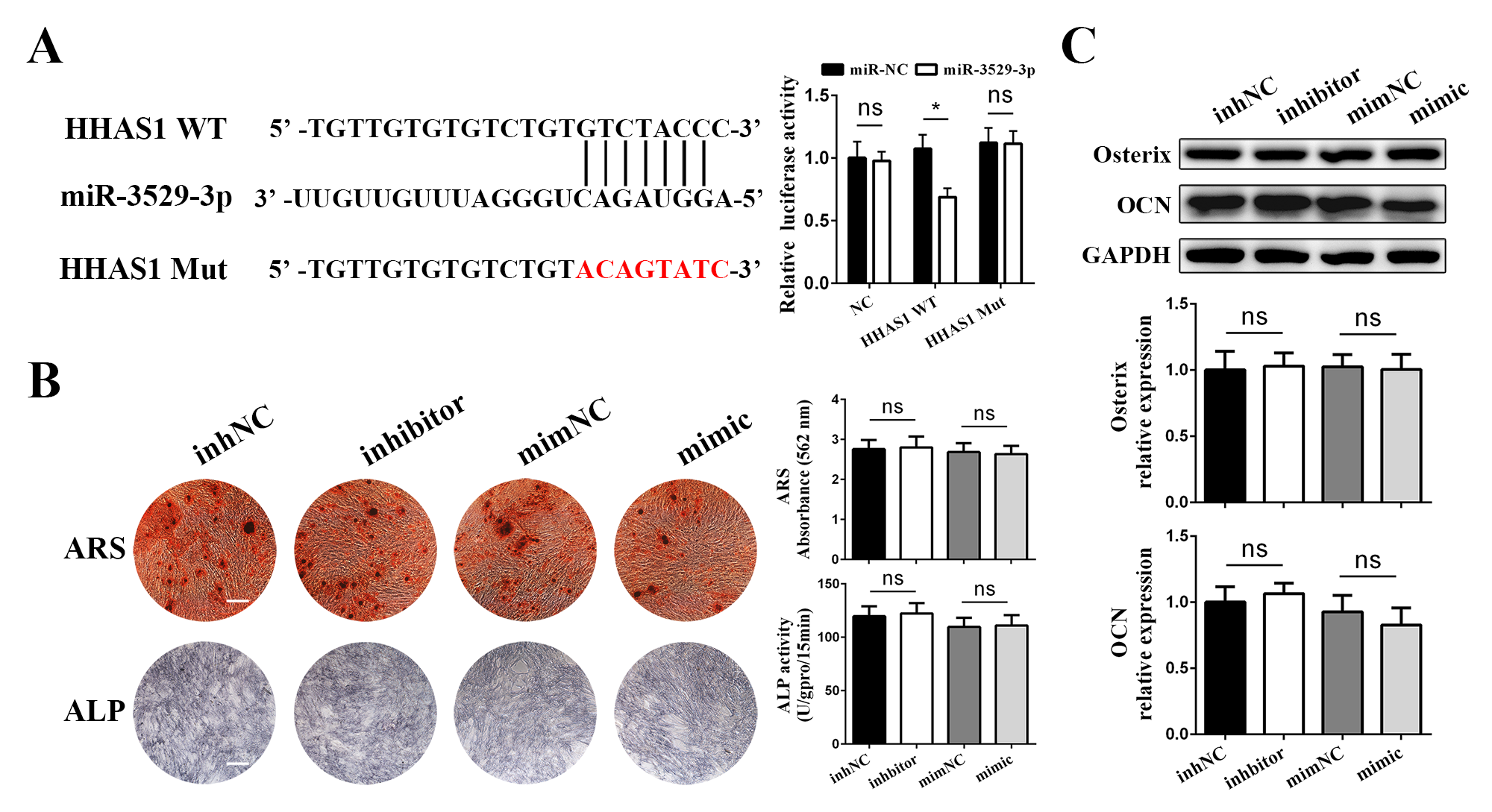

Supplement: Supplementary file 4 — Supporting information [file CTM2-11-e429-s003.tif]

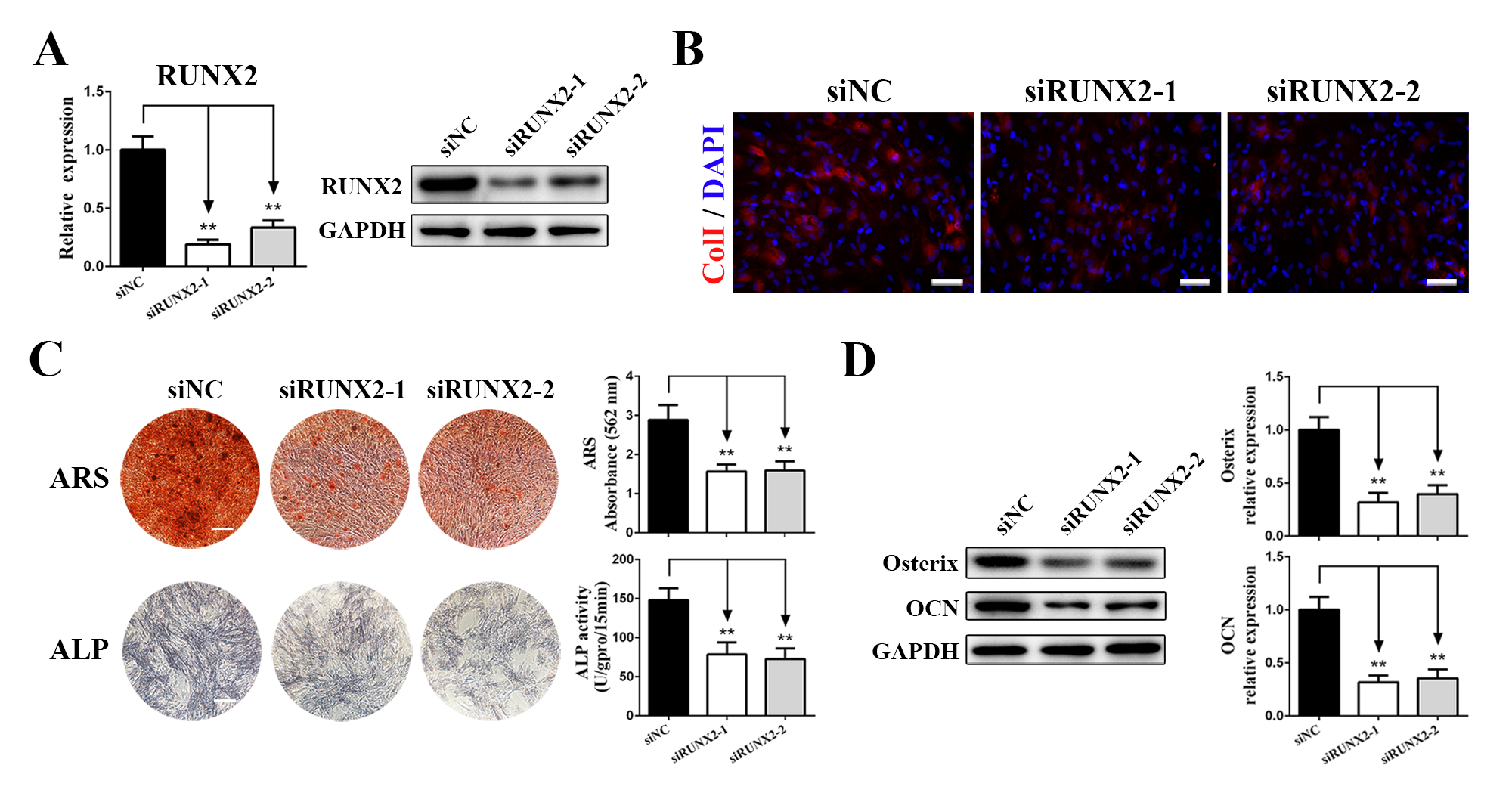

Supplement: Supplementary file 5 — Supporting information [file CTM2-11-e429-s006.tif]

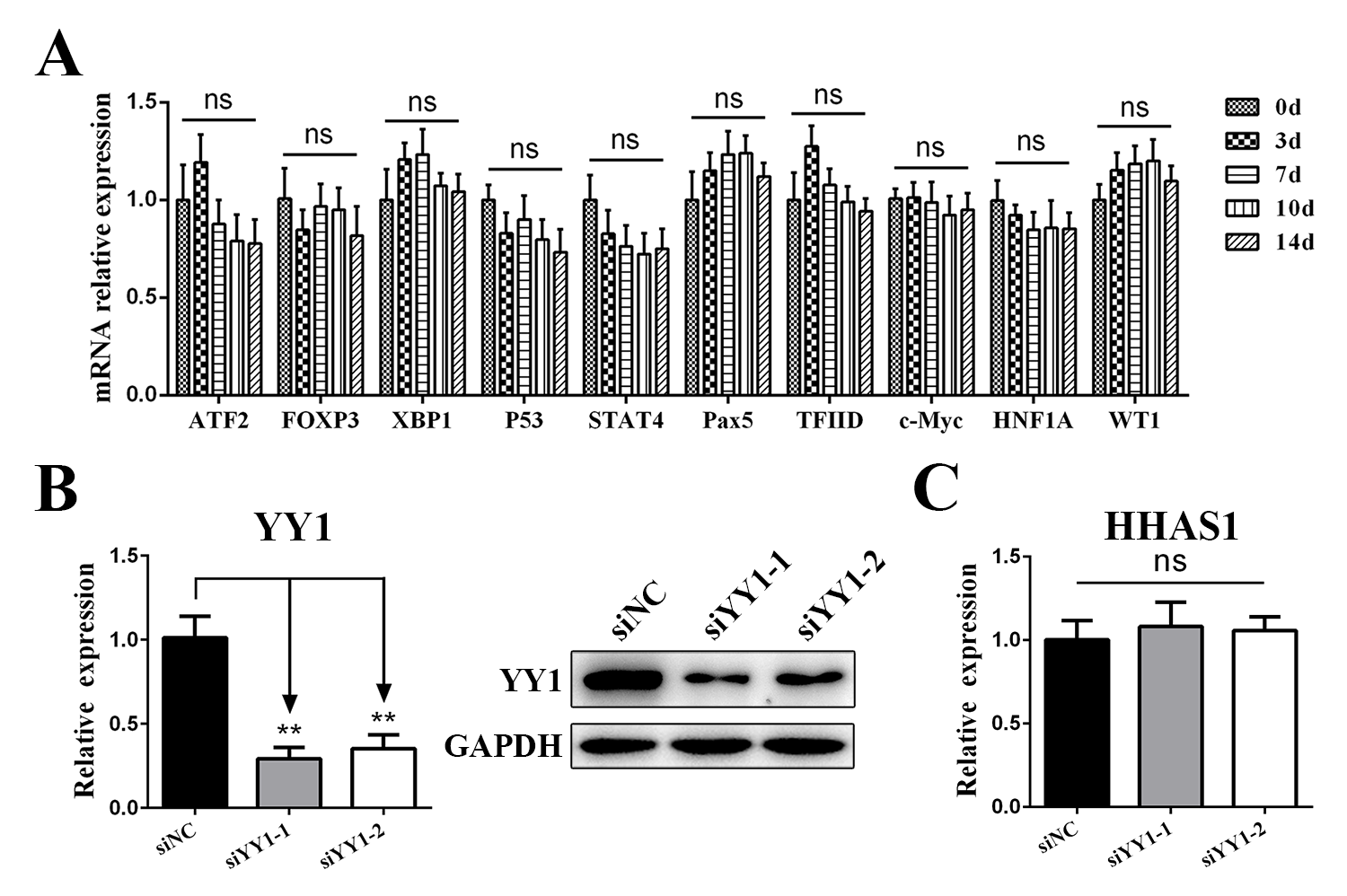

Supplement: Supplementary file 6 — Supporting information [file CTM2-11-e429-s001.tif]
