# Supplementary material for: IRF2‐mediated upregulation of lncRNA HHAS1 facilitates the osteogenic differentiation of bone marrow‐derived mesenchymal stem cells by acting as a competing endogenous RNA
Source: Clin Transl Med. 2021 Jun 20;11(6):e429. doi: 10.1002/ctm2.429 (PMC8214856; doi:10.1002/ctm2.429)
Supplement: Supplementary file 7 — Supporting information [file CTM2-11-e429-s007.doc]

**Table S1.** Primers used for qRT-PCR

| **Gene** | **Forward primer (5’-3’)** | **Reverse primer (5’-3’)** |
| --- | --- | --- |
| GAPDH | GGAGCGAGATCCCTCCAAAAT | GGCTGTTGTCATACTTCTCATGG |
| HHAS1 | TGCCTACAACCAGACTGACAGC | TCCTCTGCTTCTTCAGCCTCCAA |
| Osterix | CCTCTGCGGGACTCAACAAC | AGCCCATTAGTGCTTGTAAAGG |
| OCN | CACTCCTCGCCCTATTGGC | CACTCCTCGCCCTATTGGC |
| U6 | CTCGCTTCGGCAGCACA | AACGCTTCACGAATTTGCGT |
| MALAT1 | GTCATAACCAGCCTGGCAGT | CGAAACATTGGCACACAGCA |
| GAS5 | AGGTATGGAGAGTCGGCTTG | GCATGCTTGCTTGTTGTGGT |
| RUNX2 | TCAACGATCTGAGATTTGTGGG | GGGGAGGATTTGTGAAGACGG |
| IRF2 | CATGCGGCTAGACATGGGTG | GCTTTCCTGTATGGATTGCCC |
| YY1 | ACGGCTTCGAGGATCAGATTC | TGACCAGCGTTTGTTCAATGT |
| miR-204-5p | TCGCCGATTCCCTTTGTCATCCT | CTCAACTGGTGTCGTGGAGTCGGC |
| miR-3529-3p | TCGCCGAAACAACAAAAUCACUAGU | CTCAACTGGTGTCGTGGAGTCGGC |
| miR-204-5p-RT | CTCAACTGGTGTCGTGGAGTCGGCAATTCAGTTGAGAGGCATAG | |
| miR-3529-3p-RT | CTCAACTGGTGTCGTGGAGTCGGCAATTCAGTTGAGTGGAAGAC | |

**Table S2.** Primers used for 3’ and 5’ RACE assays

| **Oligo** | **Sequence (5’-3’)** |
| --- | --- |
| 3’ GSP1 | GATTACGCCAAGCTTACCCTTGCCTACAACCAGACTGACAGC |
| 3’ GSP2 | GATTACGCCAAGCTTACCTTAAAAGCCCCCTTGGAGGCTG |
| 5’ GSP1 | GATTACGCCAAGCTTGAATGGAGCCTGGGCAGGACTTCAG |
| FLP-F | GCGGCGGGGAGGGGCGGCTA |
| FLP-R | CAAAACACAAAATAATTTTATTTC |

**Table S3.** siRNAs used for RNA interference

| **Target Gene** | **siRNA name** | **siRNA sequence (5’-3’)** |
| --- | --- | --- |
| HHAS1 | siHHAS1-1 | GCACCAAUGCAUCUUGUAUTT |
| HHAS1 | siHHAS1-2 | GGUUAUUUCUUUAGAUUAA |
| RUNX2 | siRUNX2-1 | GGUCUGUUAUCAUCAAUAATT |
| RUNX2 | siRUNX2-2 | GCAAAGACAUAGUCAGCUATT |
| IRF2 | siIRF2-1 | GCGGUCCUGACUUCAACUATT |
| IRF2 | siIRF2-2 | GCUACUCACUCAAGUUCAATT |
| YY1 | siYY1-1 | GACGACUACAUUGAACAAATT |
| YY1 | siYY1-2 | GGUCAUAGAUGCAGAAAUATT |
