# Supplementary material for: IRF2‐mediated upregulation of lncRNA HHAS1 facilitates the osteogenic differentiation of bone marrow‐derived mesenchymal stem cells by acting as a competing endogenous RNA
Source: Clin Transl Med. 2021 Jun 20;11(6):e429. doi: 10.1002/ctm2.429 (PMC8214856; doi:10.1002/ctm2.429)
Supplement: Supplementary file 8 — Supporting information [file CTM2-11-e429-s005.doc]

**SUPPLEMENTARY FIGURE LEGENDS**

**Figure S1**. The characteristic of BMSCs. A. Flow cytometry results showed that most of the BMSCs were positive for CD29, CD44 and CD105 but not for CD14, CD45 or HLA-DR. B. The staining of Oil red O, Alizarin Sed S and Alcian blue showed that the BMSCs possessed the capability of differentiating into osteoblasts, adipocytes and chondroblasts. n = 3.

**Figure S2**. The osteogenic differentiation process of BMSCs. A. BMSCs were cultured with osteogenic induction medium for 0, 3, 7, 10 and 14 days, and the results showed increased ARS staining, ALP staining and ALP activity during the osteogenic differentiation of BMSCs (scale bar = 200 µm), *P < 0.05, **P < 0.01.

**Figure S3**. RACE assay of HHAS1. A. The electrophoresis results of the 3’ RACE, 5’ RACE and full-length HHAS1. A nest-PCR was performed in the 3’ RACE assay. GSP = gene-specific primers, FLP = full-length primers. B. The full-length sequence of HHAS1 in BMSCs according to the results of the 3’ and 5’ RACE assays. All experiments were performed at least three independent times.

**Figure S4**. miR-3529-3p does not influence the osteogenic differentiation of BMSCs. A. The binding sites of WT HHAS1 and mutated sites in MUT HHAS1 (left). Dual-luciferase reporter assays showed that the miR-3529-3p mimic inhibited the luciferase activity of the HHAS1 WT group but not the HHAS1 MUT group (right). B. Neither the inhibitor nor mimic of miR-3529-3p impacted the ARS staining, ALP staining or ALP activity of BMSCs (scale bar = 200 µm). C. Neither the inhibitor nor mimic of miR-3529-3p influenced the protein levels of Osterix and OCN in BMSCs. The data are presented as the mean ± SD (n = 10, determined by independent-sample t-tests). All experiments were performed three independent times, ns = not statistically significant, *P < 0.05.

**Figure S5**. Knockdown of RUNX2 suppresses BMSCs osteogenesis. A. The efficiency of siRNAs targeting RUNX2 measured by qPCR (left) and western blotting (right). Both siRNAs successfully downregulated the levels of RUNX2. B. Immunofluorescence detection indicated that both siRNAs targeting HHAS1 significantly reduced the Collagen I signal in BMSCs (scale bar = 50 µm). C. Both siRNAs targeting RUNX2 significantly decreased the ARS staining, ALP staining and ALP activity of BMSCs (scale bar = 200 µm). D. Both siRNAs targeting RUNX2 successfully reduced the protein levels of Osterix and OCN in BMSCs. The data are presented as the mean ± SD (n = 10, determined by independent-sample t-tests). All experiments were performed three independent times, **P < 0.01.

**Figure S6**. YY1 does not influence HHAS1 expression. A. The mRNA levels of predicted genes in BMSCs during osteogenic differentiation; none of these genes significantly increased during BMSC osteogenesis. B. The efficiency of siRNAs targeting YY1 measured by qPCR (left) and western blotting (right). Both siRNAs successfully downregulated the levels of YY1. C. Neither siRNA targeting YY1 impacted HHAS1 expression. The data are presented as the mean ± SD (n = 10, determined by independent-sample t-tests). All experiments were performed three independent times, ns = not statistically significant, **P < 0.01.
